# COVID-19 vaccines based on viral nanoparticles displaying a conserved B-cell epitope show potent immunogenicity and a long-lasting antibody response

**DOI:** 10.3389/fmicb.2023.1117494

**Published:** 2023-04-20

**Authors:** Jessica Fernanda Affonso de Oliveira, Zhongchao Zhao, Yi Xiang, Matthew D. Shin, Kathleen Elizabeth Villaseñor, Xinyi Deng, Sourabh Shukla, Shaochen Chen, Nicole F. Steinmetz

**Affiliations:** ^1^Department of NanoEngineering, University of California, San Diego, La Jolla, CA, United States; ^2^Center for Nano-ImmunoEngineering, University of California, San Diego, La Jolla, CA, United States; ^3^Moores Cancer Center, University of California, San Diego, La Jolla, CA, United States; ^4^Department of Bioengineering, University of California, Los Angeles, Los Angeles, CA, United States; ^5^Department of Bioengineering, University of California, San Diego, La Jolla, CA, United States; ^6^Institute for Materials Discovery and Design, University of California, San Diego, La Jolla, CA, United States; ^7^Department of Radiology, University of California, San Diego, La Jolla, CA, United States; ^8^Center for Engineering in Cancer, University of California, San Diego, La Jolla, CA, United States

**Keywords:** COVID-19, SARS-CoV-2, vaccine, viral nanoparticles, B-cell epitope, CPMV, Qβ virus-like particles

## Abstract

The COVID-19 pandemic caused by SARS-CoV-2 sparked intensive research into the development of effective vaccines, 50 of which have been approved thus far, including the novel mRNA-based vaccines developed by Pfizer and Moderna. Although limiting the severity of the disease, the mRNA-based vaccines presented drawbacks, such as the cold chain requirement. Moreover, antibody levels generated by these vaccines decline significantly after 6 months. These vaccines deliver mRNA encoding the full-length spike (S) glycoprotein of SARS-CoV-2, but must be updated as new strains and variants of concern emerge, creating a demand for adjusted formulations and booster campaigns. To overcome these challenges, we have developed COVID-19 vaccine candidates based on the highly conserved SARS CoV-2, 809-826 B-cell peptide epitope (denoted 826) conjugated to cowpea mosaic virus (CPMV) nanoparticles and bacteriophage Qβ virus-like particles, both platforms have exceptional thermal stability and facilitate epitope delivery with inbuilt adjuvant activity. We evaluated two administration methods: subcutaneous injection and an implantable polymeric scaffold. Mice received a prime–boost regimen of 100 μg per dose (2 weeks apart) or a single dose of 200 μg administered as a liquid formulation, or a polymer implant. Antibody titers were evaluated longitudinally over 50 weeks. The vaccine candidates generally elicited an early Th2-biased immune response, which stimulates the production of SARS-CoV-2 neutralizing antibodies, followed by a switch to a Th1-biased response for most formulations. Exceptionally, vaccine candidate 826-CPMV (administered as prime-boost, soluble injection) elicited a balanced Th1/Th2 immune response, which is necessary to prevent pulmonary immunopathology associated with Th2 bias extremes. While the Qβ-based vaccine elicited overall higher antibody titers, the CPMV-induced antibodies had higher avidity. Regardless of the administration route and formulation, our vaccine candidates maintained high antibody titers for more than 50 weeks, confirming a potent and durable immune response against SARS-CoV-2 even after a single dose.

## Introduction

Coronavirus disease-2019 (COVID-19) caused by severe acute respiratory syndrome coronavirus-2 (SARS-CoV-2) is responsible for an ongoing pandemic and major public health crisis (Lai et al., 2020; Jalkanen et al., 2021) that has resulted millions of deaths (Dejnirattisai et al., 2021; Forgacs et al., 2021; Zhou et al., 2021; Nagpal et al., 2022). The outbreak of the disease in 2019 led to an intensive global effort to develop effective vaccines (Kowalzik et al., 2021; Mathieu et al., 2021). Most of the resulting vaccines were designed to target the receptor binding domain (RBD) or full-length spike (S) glycoprotein of SARS-CoV-2, which play a key role in viral entry (Chung et al., 2020; Shin et al., 2020; Wu et al., 2020; Dejnirattisai et al., 2021; Ortega-Rivera et al., 2021). Approved vaccines, such as mRNA-based vaccines produced by companies such as Pfizer and Moderna (Patel et al., 2022), present drawback as of need for a cold chain due to their poor stability (Burki, 2021; Sah et al., 2021; Watson et al., 2022). Furthermore, a general challenge with vaccines targeting the RBD/S-protein is the decline in the antibody response after ~6 months, resulting in the need for multiple booster doses (Goel et al., 2021; Jalkanen et al., 2021; Andrews et al., 2022a,b). Besides, they also show less efficacy against new variants of concern (Dejnirattisai et al., 2021; Goel et al., 2021; Tang et al., 2021; Hajnik et al., 2022; Andrews et al., 2022a,b), such as the delta (B.1.617.2 and AY lineages) and Omicron (B.1.1.529 and subvariants BA.2.12.1, BA.4, and BA.5) strains (Hui, 2022), resulting in the need for adjusted formulations to ensure proper coverage (Goel et al., 2021; Li, 2022). For example, Pfizer and BioNTech have recently developed a BA.4/BA.5-adapted bivalent vaccine that combines the S protein mRNAs from the Wuhan strain and Omicron subvariants BA.4 and BA.5 (Burki, 2022). Similarly, Moderna has developed a bivalent vaccine combining the S protein mRNAs from the Wuhan strain and Omicron subvariant BA.1 (Chalkias et al., 2022).

We previously described COVID-19 vaccine candidates based on plant virus and bacteriophage nanoparticles that do not require cold chain distribution and can induce neutralizing immune responses after a single dose (Shin et al., 2020; Ortega-Rivera et al., 2021). Cowpea mosaic virus (CPMV) is an icosahedral plant virus (30 nm) with a pseudo-*T* = 3 symmetry that consists of 60 copies of the 24 kDa small coat protein and 42 kDa large coat protein (Huynh et al., 2016; Szabo et al., 2022). It is stable at room temperature and in the pH range 3.5–9 (Montague et al., 2011; Madi et al., 2015). Bacteriophage Qβ is similar in size to CPMV but with *T* = 3 symmetry consisting of 180 identical coat protein copies. Both CPMV and Qβ are highly immunogenic, can act as vaccine adjuvants as well as epitope display platforms, and can be produced at scale by propagation in plants or bacteria, respectively. Their size makes them ideal for enhanced antigen-presenting cell uptake and lymph node retention, significantly bolstering the immune system’s response to a conjugated antigen (Qian Wang et al., 2002; Lebel et al., 2015). Several Qβ-based vaccine candidates have already been tested in clinical trials (Bachmann and Jennings, 2010; Chariou et al., 2020; Mohsen et al., 2020).

Our viral nanoparticle (VNP)-based vaccine candidates were formulated to display B-cell epitopes from SARS-CoV-2, which are highly conserved among its variants. We screened 13 peptide epitope candidates, originally identified from convalescent sera from recovered COVID-19 patients, and identified three target epitopes (570, 636, and 826) to be suitable for vaccine design. In particular, B-cell peptide epitope 809–826 from the SARS-CoV-2 S2 glycoprotein domain (PSKPSKRSFIEDLLFNKV, denoted 826) was shown to be a potent target antigen and when displayed on CPMV or Qβ particles elicited neutralizing antibody responses against SARS-CoV-2 (Ortega-Rivera et al., 2021). The epitope 826 is highly conserved among SARS-CoV-2 variants. Vaccines that target B-cell epitopes generate more specific neutralizing antibodies compared to the broad spectrum of antibodies generated by immunization with full-length proteins, and therefore hold potential to overcome risk of antibody-dependent enhancement (Wang et al., 2016; Lee et al., 2020) or generation of non-neutralizing but interfering antibodies (Weidenbacher et al., 2022; Zanella et al., 2022).

The initiation and maintenance of a sustainable immune response against SARS-CoV-2 often requires multiple doses or prolonged exposure to the antigen (Bobbala et al., 2018). Sustained release implants therefore hold potential to alleviate the need for repeat administrations therefore enhancing patient compliance and increase the vaccine completion rate (Lee et al., 2019). Therefore, in this work, we compared the efficacy of prime–boost vs. single administration of soluble COVID-19 vaccine candidates as well as implantable polymeric formulations. The latter provide a scaffold for sustained delivery of the CPMV and Qβ-based vaccines and were fabricated using an in-house 3D printer. We used gelatin methacrylate (GelMA) as a natural matrix material due to its combination of biocompatibility and versatility during light-mediated fabrication. We were specifically interested to study the longitudinal immune responses against SARS-CoV-2 and the Omicron (B.1.1.529) variant and monitored antibody titers, Th1/Th2 bias, and antibody avidity over 50 weeks (almost 1 year).

## Methods

### Production and purification of CPMV and Qβ particles

CPMV was propagated by mechanical inoculation of black-eyed pea no. 5 plants (*Vigna unguiculata*), followed by isolation and purification as previously reported (Wellink, 1998; Nkanga et al., 2022). Briefly, around 100 g of frozen leaf tissue was homogenized in 300 ml of 0.1 M KP buffer (potassium phosphate, pH 7.0), filtered using Miracloth (Millipore, cat. no 475855), and centrifuged (18,500 g, 20 min, 4°C) to remove plant debris. The supernatant was extracted with 1:1 chloroform:1-butanol, and the aqueous phase was mixed with 0.2 M NaCl and 10% (w/v) PEG 8000 for CPMV precipitation. The mixture was centrifuged (30,000 g, 15 min, 4°C), and the pellet was resuspended in 0.1 M KP buffer (pH7). To remove aggregates, the suspension was centrifuged (13,500 g, 15 min, 4°C) and the supernatant was purified on a 10–40% (w/v) sucrose gradient ultracentrifugation (28,000 rpm, 2.5 h, 4°C) using an Optima L-90 K centrifuge with rotor type SW28 rotor (Beckman Coulter, Brea, CA, United States). The light-scattering CPMV layer collected and pelleted by ultracentrifugation (42,000 rpm, 2.5 h, 4°C) using an Optima L-90 K centrifuge with rotor type 50.2 Ti (Beckman Coulter, Brea, CA, United States). The quantity of CPMV particles was determined by UV spectroscopy at 260 nm (εCPMV = 8.1 ml^−1^). Pure CPMV particles were stored in 0.1 M potassium phosphate (KP) buffer at pH 7.0.

Bacteriophage Qβ virus-like particles (VLPs) were produced in *Escherichia coli* and purified as previously described (Ortega-Rivera et al., 2021). Briefly, 200 μl of a frozen stock of transformed competent BL21(DE3) *E. coli* cells (New England Biolabs) was cultured in 50 ml of Magic Media (Thermo Scientific, K6803) containing 25 μg/ml streptomycin (Sigma-Aldrich) and 50 μg/ml kanamycin (Gold Biotechnology) for 18 h at 37°C to saturation, shaking at 250 rpm (Ortega-Rivera et al., 2021). 50 ml of this culture was then added to 1 l Magic Media and incubated at 37°C for another 24 h, shaking at 300 rpm. Cells were then collected by centrifugation at 1,500 ×*g* and frozen at −80°C overnight. The cell pellets were resuspended in lysis buffer (GoldBio Cat# GB-177) on ice and lysed using a probe sonicator for 10 min. The lysate was centrifuged at 5,000 × *g*, the supernatant was collected, and the Qβ particles were precipitated by adding 10% (w/v) PEG8000 (Thermo Fisher Scientific) at 4°C for 12 h on a platform shaker. The precipitated fraction was pelleted by centrifugation at 5,000 ×*g* and dissolved in 40 ml PBS before extraction with a 1:1 v/v butanol/chloroform. The aqueous fraction was collected by centrifugation as above (at 5,000 ×*g*) and Qβ particles were purified on a 10–40% (w/v) sucrose gradient ultracentrifugation (28,000 rpm, 2.5 h, 4°C) using an Optima L-90 K centrifuge with rotor type SW28 rotor (Beckman Coulter). The light-scattering Qβ layer was collected and pelleted by ultracentrifugation at (42,000 rpm, 2.5 h, 4°C) using an Optima L-90 K centrifuge with rotor type 50.2 Ti (Beckman Coulter). The purified Qβ particles were resuspended in PBS and stored at 4°C until further use. Qβ particles were quantified using a Pierce BCA assay kit (Thermo Fisher Scientific).

### Bioconjugation of peptide 826

Peptide 826 (original ID S21P2) is a B-cell epitope spanning residues 809–826 (PSKPSKRSFIEDLLFNKV) of the highly conserved S2 domain of the SARS-CoV-2 S-protein (Ortega-Rivera et al., 2021). When conjugated to a carrier, this peptide can induce the neutralization of SARS-CoV-2. Peptide 826 was synthesized by GenScript, including an N-terminal CGGG linker for conjugation to CPMV or Qβ using a two-step method as previously described (Ortega-Rivera et al., 2021; Shin et al., 2021).

### Characterization of CPMV and Qβ vaccine candidates

For agarose gel electrophoresis, 1.2% (w/v) agarose gels stained with GelRed (Gold Biotechnologies) were loaded with 10 μg CPMV or 826-CPMV followed by separation in TAE buffer for 30 min at 120 V and 400 mA. Gels were imaged under UV light to visualize the RNA and stained with Coomassie Brilliant Blue G-250 (0.25% w/v) and imaged under white light to detect the protein on a FluorChem R system (ProteinSimple). For polyacrylamide gel electrophoresis, 10 μg of native particles or corresponding 826-conjugates was mixed with 4× sample buffer (Thermo Fisher Scientific) and 10× NuPAGE reducing agent (Invitrogen) at 95°C for 5 min before separation in NuPAGE 4–12% Bis Tris protein gels (Invitrogen) in 3-(*N*-morpholino)propanesulfonic acid (MOPS) buffer (Thermo Fisher Scientific) at 200 V and 120 mA for 40 min. The gel was stained with GelCode Blue Safe protein stain and visualized under white light to image both the CPMV and Qβ coat proteins on a FluorChem R system. For dynamic light scattering (DLS), the hydrodynamic diameter of CPMV, Qβ, and the conjugated particles was assessed using a Zetasizer Nano ZSP/Zen5600 (Malvern Panalytical) at 25°C with three measurements per 1 mg/ml sample. The particle diameter was calculated as the weighted mean of the intensity distribution. For transmission electron microscopy (TEM), formvar carbon film-coated TEM supports with 400-mesh hexagonal copper grids (VWR International) were rendered more hydrophilic using the PELCO easiGlow operating system. CPMV, Qβ, and the conjugated particles (0.1 mg ml^−1^ in deionized water) were loaded onto the grids and stained with 2% (w/v) uranyl acetate (Agar Scientific). The samples were imaged using a FEI Tecnai Spirit G2 BioTWIN TEM at 80 kV.

### Fabrication of the implantable polymeric scaffold

GelMA was synthesized and characterized as previously described (Soman et al., 2013). Polyethylene glycol diacrylate (Mn 700, PEGDA700) was purchased from Sigma-Aldrich. Lithium phenyl-2,4,6-trimethylbenzoylphosphinate (LAP) was synthesized as previously described (Fairbanks et al., 2009) and was stored under argon at 4°C for use as a photo-initiator. The bioink for scaffold printing comprised 4% (w/v) GelMA, 0.1% (v/v) PEGDA, and 0.6% (w/v) LAP in Dulbecco’s PBS (DPBS, Gibco). For the CPMV/Qβ-laden scaffolds, CPMV/Qβ were first resuspended in DPBS to the designated concentration, and the suspension was used to prepare the bioink. The scaffold was printed layer-by-layer using an in-house digital light projection (DLP) 3D bioprinter, which consists of a blue light source (405 nm), a digital micromirror array device for optical pattern generation, a set of projection optics, a motorized stage to guide the fabrication of each layer, and a computer control system. For each layer, a user-defined blue light pattern was projected on the bioink reservoir and only the illuminated area was polymerized. After one layer was polymerized, the stage was lifted by the designated layer thickness, where bioink refilled the gap and allowed the fabrication of the subsequent layer.

### Characterization of the implantable polymeric scaffold

As-printed scaffolds were lyophilized and manually compressed into a thin film for Fourier transform infrared (FTIR) spectroscopy (32 scans) using the PerkinElmer Spectrum Two FT-IR device with a universal attenuated total reflectance (UATR) crystal. A MicroSquisher (CellScale) was used to measure the Young’s modulus of the scaffolds in compression mode. For each the CPMV/Qβ formulation, cylinders with a height of 1.5 mm (one layer) and a diameter of 1.5 mm were printed with the same material as the implants to accommodate the setting of the MicroSquisher. The data were collected in displacement mode using the ramp function. The compression magnitude was set at 20% of the sample height and the loading and recovery duration was 0.125 s/μm. As-printed scaffolds were also lyophilized and manually sectioned to facilitate the imaging of their internal microstructure by scanning electron microscopy (SEM). The samples were mounted on the stage with conductive tape and coated with iridium using an Emitech K575X sputter coater. The samples were imaged using an FEI Apreo HiVac device operating at 3 kV.

### Quantification of CPMV and Qβ *in vitro* release

CPMV and Qβ implants were placed in 1.7-ml Eppendorf tubes containing 1 ml PBS and incubated at 37°C for 30 days, shaking at 100 rpm. We collected 1 ml aliquots of sample after 1, 3, 7, 10, 14, 20, and 30 days, and added 1 ml of fresh PBS to allow continuous particle release. CPMV particles released from implants were quantified using a CPMV ELISA kit (CD Biosciences) and a standard curve based on 0.05, 0.1, 0.15, 0.25, and 0.5 μg/ml pure samples. All released CPMV samples were diluted 100-fold in PBS before testing. MaxiSorp 96-well plates (Thermo Fisher Scientific) were coated with 100 μl of capture antibody overnight before washing five times with 200 μl PBS containing 0.05% (v/v) Tween-20 (PBST). The CPMV standards and released CPMV samples (100 μl) were added to the wells and incubated at room temperature for 2.5 h. The plate was then washed seven times with 200 μl PBST, and 100 μl of the conjugating enzyme was added to each well and incubated at room temperature for 2.5 h. After another seven washes as above, 100 μl of detection substrate was added to each well and incubated at room temperature for 10 min. Finally, we added 50 μl of stop solution to each well. Absorbance was recorded at 405 nm using a Tecan plate reader and values were checked against the standard curve to determine the quantity of CPMV particles in each sample. Qβ released from implants was quantified by SDS-PAGE by comparing samples with 10, 20, 30, 40, and 50 μg/ml of purified Qβ loaded onto NuPAGE 4–12% Bis Tris protein gels and separated at 200 V and 120 mA for 35 min. Gels were stained with Coomassie Brilliant Blue and imaged using the FluorChem R system as above, followed by quantification using ImageJ software.

### Immunization of mice

All animal experiments were carried out in accordance with University of California San Diego’s Institutional Animal Care and Use Committee (IACUC). All animals used in this study were 7–8-week-old male BALB/c mice obtained from the Jackson Laboratory (strain #000651). For subcutaneous (s.c.) injection with liquid formulations, each vaccine candidate was prepared in sterile PBS (Corning, 21-040-CV). We compared a prime–boost regimen to a single dose. For the prime–boost regimen, the candidates were prepared at a concentration of 1 mg/ml and we administered two s.c. doses of 100 μl (100 μg) injected 2 weeks apart. For the single-dose regimen, the candidates were prepared at a concentration of 2 mg/ml and we administered one s.c. dose of 100 μl (200 μg). For immunization using the slow-release implants, a single-dose implant containing 200 μg of the vaccine candidate was surgically implanted s.c. behind the neck. Five mice were assigned to each group. Blood was collected in lithium–heparin-treated tubes (Thomas Scientific) by retro-orbital bleeding before immunization (week 0) and then every 2 weeks from weeks 2 to 50 post-immunization. Plasma was collected by centrifugation at 2000 g for 10 min at 4°C and was stored at −80°C.

### IgG titers against the peptide and S-protein

End-point IgG titers against the 826-peptide epitope (CGGGPSKPSKRSFIEDLLFNKV) displayed on the CPMV or Qβ vaccine candidates were determined by ELISA. We coated 96-well, maleimide-activated plates (Thermo Fisher Scientific) with 150 μl/well of the peptide (20 μg/ml in coating buffer: 0.1 M sodium phosphate, 0.15 M sodium chloride, 10 mM EDTA, pH 7.2) overnight at 4°C. After three washes in PBST (0.05% (v/v) Tween-20 in PBS), the plates were blocked for 1 h at room temperature with 200 μl/well of 10 μg/ml l-cysteine (Sigma-Aldrich). After washing as above, plasma samples from immunized animals (serially diluted two-fold in coating buffer) were added and incubated for 1 h at room temperature. After further washing, we added the horseradish peroxidase (HRP)-labeled goat anti-mouse IgG secondary antibody (Thermo Fisher Scientific) diluted 1:5000 in PBST (100 μl/well) and incubated for 1 h at room temperature. After a final washing step, the signal was developed with 100 μl/well of 1-Step Ultra TMB-ELISA substrate solution (3,3′,5,5′-tetramethylbenzidine, Thermo Fisher Scientific) for 2 min at room temperature and quenched with 50 μl of 2 N sulfuric acid (Spectrum Chemical). Absorbance was read at 450 nm using an Infinite 200 Pro microplate reader and i-control software (Tecan, Männedorf, Switzerland). The IgG titer against SARS-CoV-2 and SARS-CoV-2 B.1.1.529 (Omicron) S protein was determined as described above for the peptide but using 96-well nickel-activated plates (Thermo Fisher Scientific) coated with 200 ng of His_6_-tagged S-protein (GenScript Biotech) or Omicron B.1.1.529 S1 protein (Sino Biological, 40,591-V08H41) per well. Plasma samples were diluted 1:1000 in PBS. The same secondary antibody dilution and substrate as above were used to detect the signal. The absorbance was read at 450 nm on a Tecan microplate reader. The end-point antibody titers were defined as the reciprocal serum dilution at which the absorbance exceeded twice the background value (blank wells without plasma sample).

### Antibody isotyping

We followed the ELISA protocol described above, but samples were diluted 1:400 in coating buffer before testing. Secondary HRP-labeled goat anti-mouse antibodies specific for IgG1 (Invitrogen PA174421), IgG2a (Thermo Scientific A-10685), IgG2b (Abcam ab97250), and IgM (Abcam ab97230) were diluted 1:5000. The IgG2a/IgG1 ratio was reported for each group, and a ratio higher than 1 was considered as a Th1 response.

### Avidity ELISA

The IgG antibody avidity against the SARS-CoV-2 and SARS-CoV-2 B.1.1.529 (Omicron) S proteins was evaluated by ELISA using nickel-coated plates as described above. The plates were coated with 1 μg/ml of SARS-CoV-2 or Omicron S protein (100 μl/well) in PBS overnight at 4°C and then washed three times in PBST. Mouse serum was diluted 1:20 before adding to the plates, followed by a threefold serial dilution series (100 μl/well) in blocking buffer (1% (w/v) bovine serum albumin in PBS). A negative control was added in the last column (blocking buffer without plasma). The plates were incubated for 1 h at room temperature before washing once in PBST then 3 × 5 min in PBST or PBST containing 7 M urea. We then added an HRP-labeled goat anti-mouse IgG secondary antibody (Thermo Fisher Scientific) diluted 1:5000 in PBST (100 μl/well) and incubated at room temperature for 1 h. The same secondary antibody dilution and substrate as above were used to detect the signal. The absorbance was read at 450 nm on a Tecan microplate reader. The end-point antibody titers were defined as the reciprocal serum dilution at which the absorbance exceeded twice the background value (blank wells without plasma sample). IgG avidity was expressed as the avidity index (AI) and calculated using the following formula: AI = (mean OD of urea-treated serum/mean OD urea-untreated serum) × 100%.

### Statistical analysis

Data were processed and analyzed using GraphPad Prism v8.0.2 (GraphPad Software, San Diego, CA, United States), unless otherwise indicated. Depending on the datasets, data were statistically compared by one-way analysis of variance (ANOVA) followed by Tukey’s multiple comparison test or two-way ANOVA using pairwise multiple comparison followed by Tukey’s multiple comparison test. Asterisks in figures indicate significant differences between groups (*p < 0.05; **p < 0.01; ***p < 0.001; ****p < 0.0001). Table with all statistics data can be found in Support Information Excel Spreadsheet.

## Results and discussion

### Characterization of VNP/VLP-based vaccine candidates

**Figure 1:**
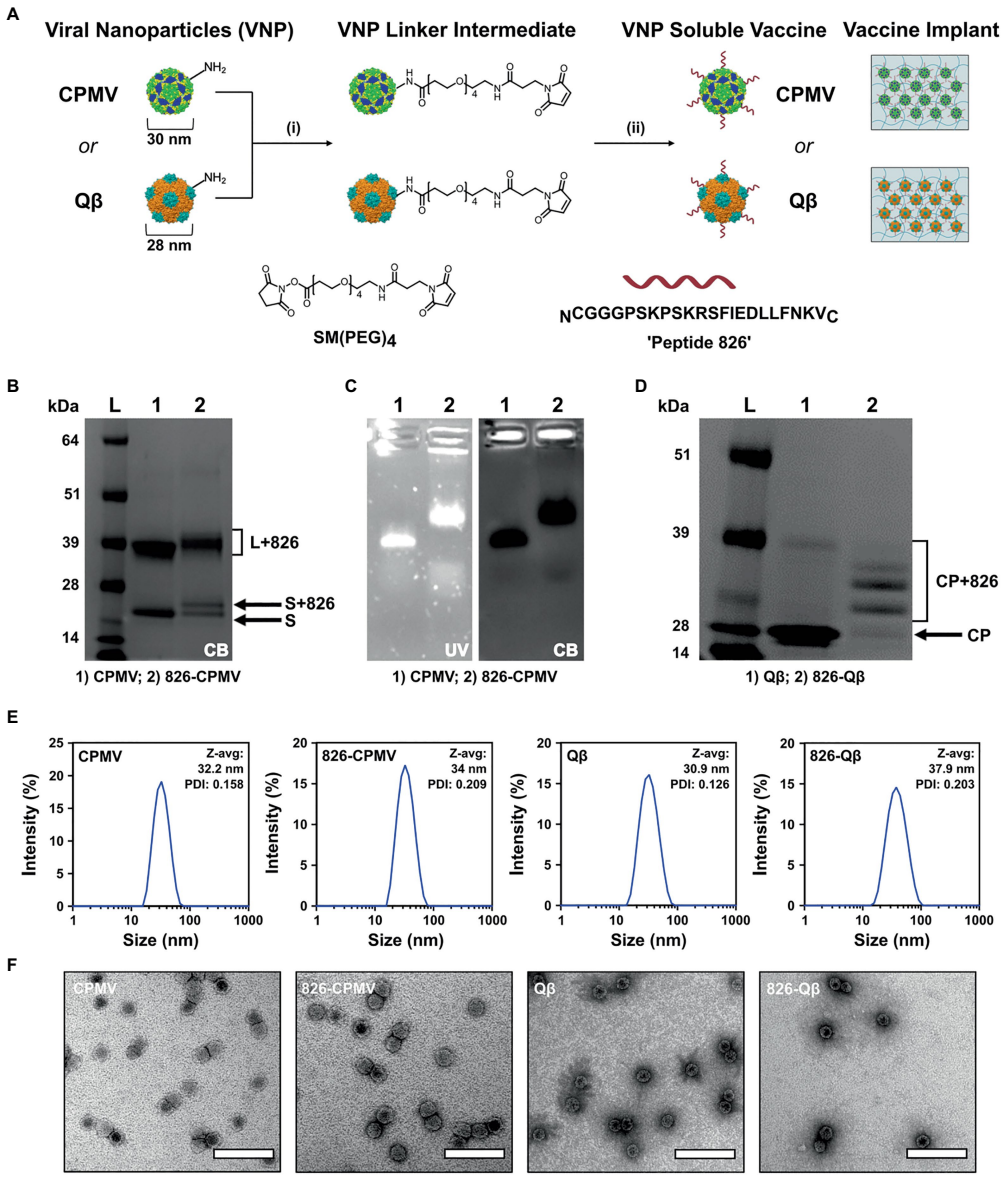
Characterization of 826-CPMV and 826-Qβ particles. **(A)** Conjugation scheme for conjugate vaccines and their loading in implants, showing the structure of the SM(PEG)_4_ heterobifunctional linker and epitope 826. **(B)** SDS-PAGE analysis of 826-CPMV showing the conjugated protein bands (S = small coat protein, L = large coat protein, +826 = conjugated peptide). **(C)** Analysis of 826-CPMV by 1.2% (w/v) agarose gel electrophoresis (gels stained with GelRed and Coomassie Brilliant blue). **(D)** SDS-PAGE analysis of 826-Qβ showing the conjugated protein bands. **(E)** DLS spectra with Z-avg (d, nm) and polydispersity index (PDI) values for the peptide conjugates. **(F)** TEM images of the peptide conjugates negatively stained with 2% (w/v) uranyl acetate (scale bar = 100 nm).

CPMV nanoparticles (VNP) and Qβ VLPs are versatile platforms that can be produced in bulk by propagation in plants or fermentation in bacterial cultures, respectively, and then be engineered by chemical conjugation to display epitopes such as peptide 826. Bacterial fermentation is a standard method for the production of biologics so Qβ VLPs are more readily translated to a cGMP platform approved for clinical trials (Bachmann and Jennings, 2010; Chariou et al., 2020; Mohsen et al., 2020), while plant molecular farming companies have been established the methods require more specialized with few Contract Research Organization (CRO) available. The CPMV and Qβ viral capsids host solvent-exposed lysine residues suitable for chemical conjugation, and we used these for the multivalent display of epitope 826 (Figure 1A). Accordingly, we synthesized 826-CPMV and 826-Qβ vaccine candidates (Ortega-Rivera et al., 2021) and characterized them by electrophoresis, DLS and TEM (Figures 1B–F). The chemistry was as previously established (Ortega-Rivera et al., 2021; Nkanga et al., 2022) by targeting the solvent-exposed lysine side chains using a bivalent NHS-PEG-maleimide (SM-PEG_4_) linker which connects to the 826 peptide by an added terminal cysteine.

Conjugation of the peptide was confirmed by SDS-PAGE and agarose gel electrophoresis. SDS-PAGE showed the CPMV and Qβ specific coat protein bands at 24 kDa and 42 kDa for CPMV and ∼14 kDa for Qβ—additional higher molecular weight bands corresponding to the coat protein coupled to the 826 peptide (MW = 2379.74) were also apparent (Figures 1B,D). Densitometric analysis indicated that ~50–55% of the CPMV coat proteins were modified (Figure 1B), in agreement with previous reported values (Ortega-Rivera et al., 2021). Similarly, SDS-PAGE and densitometric analysis revealed that ~60% of the Qβ coat proteins were modified (Figure 1D), in agreement with previously reported values (Ortega-Rivera et al., 2021). Native agarose gel electrophoresis revealed an increase in electrophoretic mobility of 826-CPMV vs. CPMV toward the anode following peptide conjugation (as a result of lysine modification). The colocalization of nucleic acid and protein staining confirmed that the particles were intact (Figure 1C). DLS revealed that the 826-CPMV particles (Z_av_ ~ 34 nm, PDI = 0.209) were larger than native CPMV (Z_av_ ~ 32.2 nm, PDI = 0.158); the same observation was made for Qβ with Z_av_ ~ 37.9 nm (PDI = 0.203) for 826-Qβ particles vs. Z_av_ ~ 30.9 nm (PDI = 0.126) for unmodified Qβ (Figure 1E). The hydrodynamic diameter of CPMV and Qβ vaccine candidates (Ortega-Rivera et al., 2021), as well as other particles displaying SARS-CoV-2 S-protein epitopes (Li et al., 2020), has been reported to increase following the conjugation of peptides. The more pronounced “swelling effect” in Qβ particles may reflect the greater number of displayed peptides. TEM confirmed that the 826-CPMV and 826-Qβ particles remained intact after conjugation (Figure 1F). Together, these results indicated that CPMV and Qβ particles can be efficiently conjugated to epitope 826 and retain their structural integrity.

### Bioprinting and characterization of VNP-laden polymeric scaffolds

**Figure 2:**
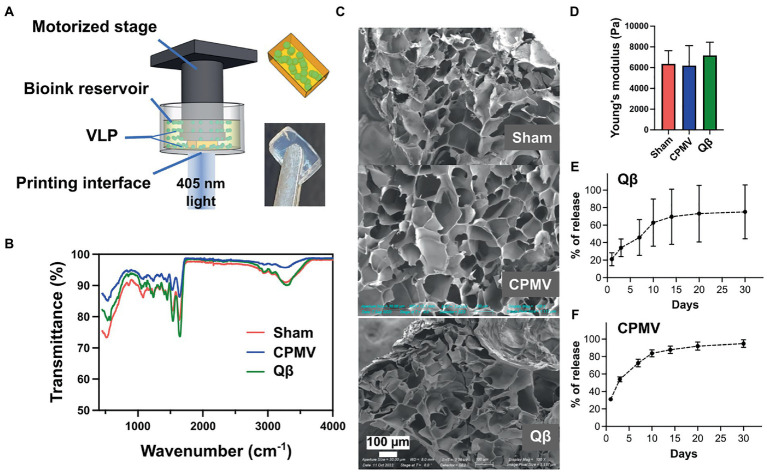
Bioprinting and characterization of CPMV and Qβ implants. **(A)** Schematic diagram of the digital light processing (DLP) bioprinting approach. Characterization of empty (sham), CPMV and Qβ implants by **(B)** FITR spectroscopy, **(C)** SEM and **(D)** the measurement of Young’s modulus showed no significant differences. Release profiles of printed **(E)** Qβ and **(F)** CPMV implants *in vitro*.

The continuous release of antigens increases the duration of interaction between the antigen and the immune system, thus amplifying the humoral response and boosting vaccine efficacy (Bobbala et al., 2018). We therefore theorized that an implant providing a continuous supply of VNPs may yield a better immune response than individual doses (Hou et al., 2021). Accordingly, we used DLP printing (Figure 2A) to fabricate implantable polymeric scaffolds containing VNPs, allowing their continual release *in vivo*. The VNPs were confined within the polymeric network of the scaffold and released after implantation by Fick’s diffusion and biodegradation of the matrix material (Sun et al., 2020). The release rate of VNPs is defined and tunable by the concentration of the VNPs in the scaffold, their aspect ratio, and the degree of polymerization of the scaffold matrix (Caccavo et al., 2015). A 4% (w/v) GelMA matrix was prepared and loaded with VNPs to achieve a final concentration of 2.381 mg/ml, allowing a sustained, moderate release rate. The bioink also contained 0.1% (w/v) PEGDA to enhance printability and ease of handling. Each 1.5-mm layer was exposed to light for 30 s (79.4 mW/cm^2^) to ensure sufficient polymerization. Each scaffold, with a payload of 200 μg VNPs, was printed as two layers with a 4 × 7 mm^2^ rectangular shape.

FTIR spectroscopy confirmed successful polymerization initiated by the DLP printing process (Figure 2B). A sharp decrease in transmittance was observed at ~1,640 cm^−1^ in all scaffolds (with and without the VNPs), representing the typical C=O stretching vibration between the methacrylate groups of polymerized GelMA. SEM images of the lyophilized scaffolds showed a porous structure resulting from the polymeric network of GelMA (Figure 2C). All scaffolds had a similar porous microstructure, indicating that the presence of VNPs did not interfere with the polymerization process. Mechanical testing demonstrated that scaffolds prepared from the same matrix material under designated printing conditions possessed a similar Young’s modulus (sham: 6370.92 ± 1262.98 Pa, CPMV-loaded: 6192.39 ± 1937.18 Pa, Qβ-loaded: 7189.61 ± 1266.63 Pa), which conformed that the degree of polymerization could be controlled (Figure 2D).

The *in vitro* release profiles of the laden scaffolds were characterized by SDS-PAGE for Qβ (Supplementary Figure S1) and ELISA for CPMV (Supplementary Figure S2) against generated standard curves. A similar trend was observed in both formulations, featuring a burst release within the first 24 h (20% of the Qβ particles and 30% of the CPMV particles), followed by gradual and continuous release over the following days (Figures 2E,F). After 2 weeks, 60% of the Qβ particles and 80% of the CPMV has been released from the scaffold.

### Immunogenicity of VLP-based vaccine candidates

The immunogenicity of CPMV and Qβ particles displaying epitope 826 was evaluated in BALB/c mice using a previously reported dosing schedule (Ortega-Rivera et al., 2021; Nkanga et al., 2022). We compared a prime–boost regimen (100 μg particles injected s.c., weeks 0 and 2) with a single dose (200 μg particles injected s.c., week 0) and polymeric implants (containing 200 μg particles, introduced s.c. behind the neck, week 0). Blood samples were collected by retro-orbital bleeding every 2 weeks for 50 weeks post-immunization and plasma was screened for antibodies against the target epitope by ELISA.

**Figure 3:**
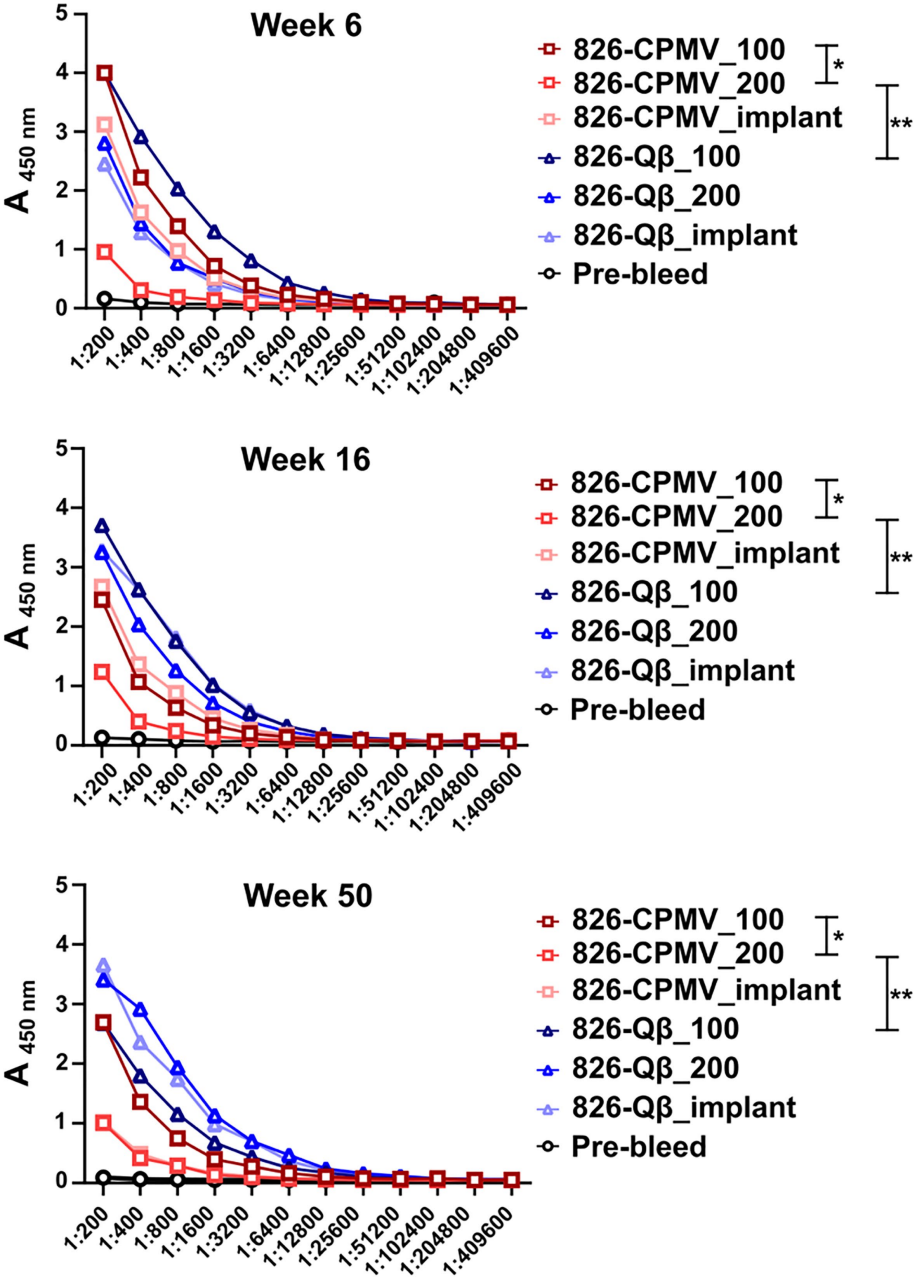
Antibody response of CPMV/Qβ vaccine candidates after s.c. administration. Mice received (Lai et al., 2020) two doses of 100 μg particles (826-CPMV_100 or 826-Qβ_100), (Jalkanen et al., 2021) one dose of 200 μg particles (826-CPMV_200 or 826-Qβ_200), or (Nagpal et al., 2022) the slow-release implant (826-CPMV_implant or 826-Qβ_implant). Antibody titers against the 826 epitope were measured by ELISA during the post-immunization period for 50 weeks. Two-way ANOVA using pairwise multiple comparison followed by Tukey’s multiple comparison test was used to compare between groups. Asterisks in the Support Information Supplementary Table S1 indicate significant differences between groups (*p < 0.05; **p < 0.01, ****p < 0.0001).

All vaccine candidates elicited antibodies against the 826 epitope and epitope-specific antibodies remained at significant levels after 50 weeks (Figure 3). Overall, the Qβ formulations demonstrated the highest titers at the 50-week time point (Figure 3; Supplementary Figure S3). Although the single-dose CPMV formulation (826-CPMV_200) elicited the lowest overall titers, they remained constant for 50 weeks. Differences in antibody titers were more apparent at week 50, with the highest titers observed in mice immunized with 826-Qβ_200 particles or the 826-Qβ_implant and the lowest in those immunized with the 826-CPMV_200 particles or the 826-CPMV_implant. The >8-fold higher titers (by week 50) of the Qβ-based vaccine may be explained by the larger number of epitopes delivered compared to CPMV; the Qβ-based vaccine displayed ~457 peptides and CPMV-based vaccine ~61 peptides (see Figure 1).

**Figure 4:**
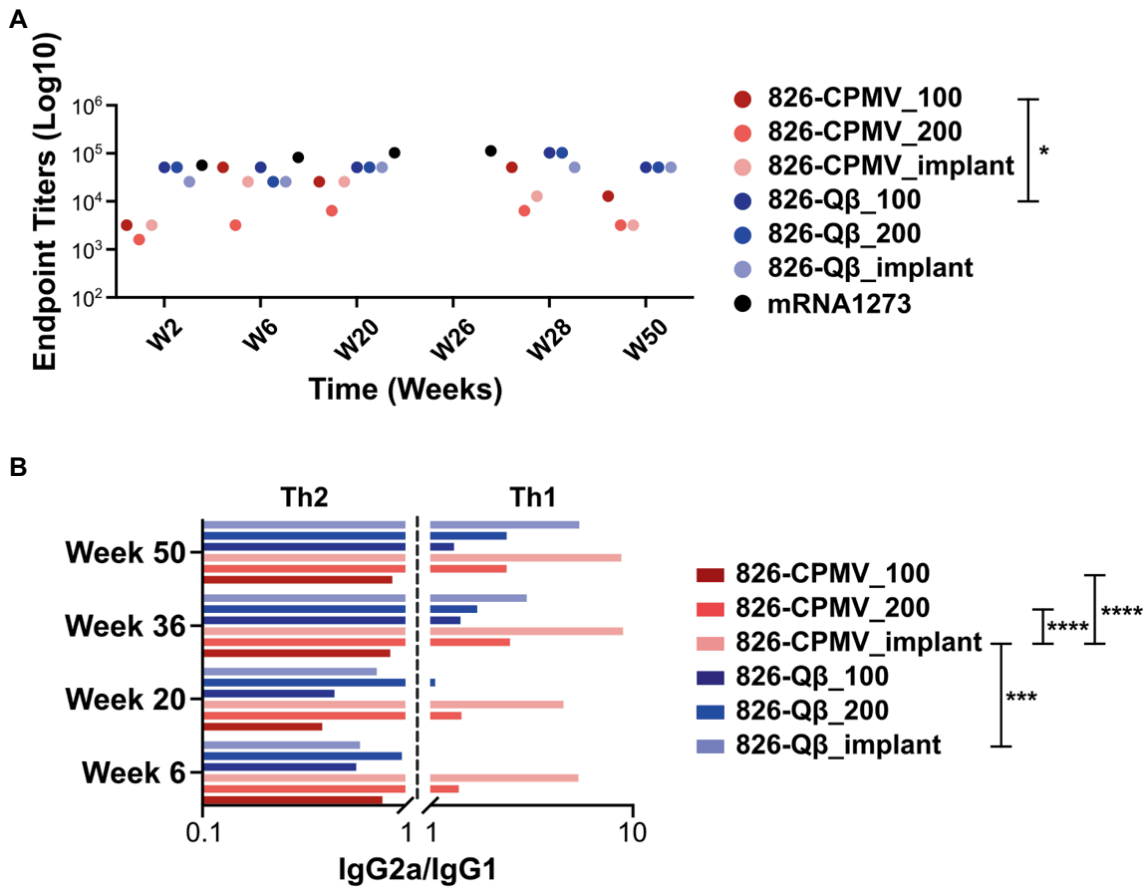
**(A)** End-point IgG titers after the prime–boost administration of CPMV/Qβ-based vaccine candidates. All candidates elicited IgG recognizing epitope 826 at 2 weeks post-immunization. Samples from mice were pooled for analysis (*n* = 5). **(B)** IgG subclass profile (IgG2a/IgG1 ratio) from weeks 4–50. A ratio < 1 is considered as a Th2-biased response and a ratio >1 is considered as a Th1-biased response. □ = mRNA-RBD; open-source data of an mRNA-RBD vaccine (Huang et al., 2021). Two-way ANOVA using pairwise multiple comparison followed by Tukey’s multiple comparison test was used to compare between groups. Asterisks in the Support Information Supplementary Tables S2, S3 indicate significant differences between groups (*p < 0.05; **p < 0.01, ****p < 0.0001).

While high end-point titers were observed for all vaccine formulations tested and maintained over 50 weeks; differences in end-point antibody titers we observed (Figure 4A): Off note, the end-point titers of the Qβ-based vaccine candidate were comparable to those reported for an open-source end point titer data of a mRNA-RBD vaccine (Huang et al., 2021). The 826-Qβ formulations showed more prominent and consistent longitudinal antibody titers than the 826-CPMV formulations, with a four-fold decrease from week 28 to week 44 then consistent titers up to week 50 (1:12,800 for 826-CPMV_100 and 1:3,200 for the other CPMV formulations). The 826-CPMV_100 formulation showed a 16-fold increase in end-point titers from weeks 2–28 (1:3,200 to 1:51,200), then a four-fold decrease to 1:12,800 at week 44. The single-dose formulation 826-CPMV_200 elicited an increase in titers at the 6- and 20-week time points but remained at ~1:3,200 throughout. The implant formulations fluctuated between 1:25,600 and 1:3,200 (week 50). Compared to the BNT162b2 and mRNA-1,273 vaccines (Collier et al., 2021), where the antibody titer declined sharply after 6 months and even more after 8 months, our formulations maintained high antibody titers for about 1 year.

While our vaccines are designed with B-cell epitopes aimed at eliciting humoral responses, induction of cellular immunity and T helper cells is another important aspect to SARS-CoV-2 vaccines (Chung et al., 2022). Antigen processing requires priming by T helper cells (Th1 and Th2, among others) to activate B cells (Ortega-Rivera et al., 2021). The choice between Th1 and Th2 responses depends on the cytokine release profile, and a balanced response is a key factor that influences the immune response (Berger, 2000; Smith et al., 2000). Th1 cells secrete IFN-γ, causing activated macrophages to induce the production of opsonizing antibodies (IgG2a/b) by B cells (Ortega-Rivera et al., 2021). Th1 responses help to control intracellular pathogens (Rosenthal and Zimmerman, 2006; Ortega-Rivera et al., 2021). In contrast, Th2 cells secrete cytokines such as IL-4, which elicit B cells to produce neutralizing antibodies (IgG1). Th2 responses are predominantly antibody-based, inducing a humoral response that protects against extracellular pathogens, allergens, and toxins (Rosenthal and Zimmerman, 2006; Ortega-Rivera et al., 2021; Chung et al., 2022). Another way to differentiate between Th1 vs. Th2 is by probing which antibody subtypes are produced. Therefore, we profiled the immunoglobulin isotypes (IgG vs. IgM) and IgG subclasses IgG1, IgG2a and IgG2b (Supplementary Figure S4). The IgG2a/IgG1 ratios indicated that the Qβ formulations initially induced a Th2-biased response which shifted to a Th1-biased response—and this was independent of the formulation, but with the implant being the most Th1-biased (Figure 4B). In contrast, the 826-CPMV_100 formulation induced a Th2-biased response at all stages (albeit more balanced at weeks 36 and 50), whereas the other CPMV formulations induced a Th1-biased response from as early as week 6. Comparing the profiles for CPMV and Qβ reveals that both the epitope and carrier determine whether Th1 or Th2 bias is established. Also, the implant formulation caused a stronger shift toward a Th1-bias.

**Figure 5:**
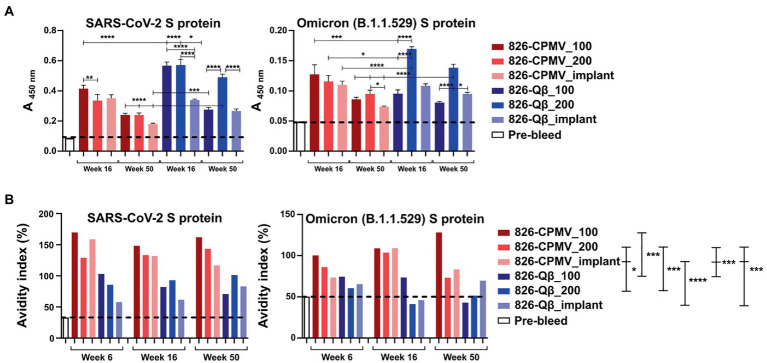
Analysis of antibody binding and avidity. **(A)** ELISA analysis of IgG binding to SARS-CoV-2 and Omicron (B1.1.529) S-proteins. Absorbance (450 nm) of pooled plasma from animals vaccinated with the different formulations. One-way analysis of variance (ANOVA) followed by Tukey’s multiple comparison test was used to compare between groups. **(B)** Avidity index of S-protein-specific IgG. The dashed line indicates the cutoff value. Two-way ANOVA using pairwise multiple comparison followed by Tukey’s multiple comparison test was used to compare between groups. Asterisks in the Support Information Supplementary Tables S4, S5 indicate significant differences between groups (*p < 0.05; **p < 0.01, ****p < 0.0001).

We then tested whether the mouse plasma, and antibodies elicited, showed specificity against the S-protein. An ELISA format was used, and we confirmed the presence of IgG specific for the SARS-CoV-2 Wuhan and Omicron S-protein variants (Figure 5A). All vaccine candidates tested positive and recognized the Wuhan and Omicron strains; there was a consistent trend of slightly decreasing titers over time, but significance was established throughout the 1-year time-course. It was noted however that the overall titers against the Omicron strain tested (B.1.1.529 S1 protein) were at least four-fold lower compared to the Wuhan strain. Consistent with the antibody titers against the peptide epitope, the Qβ formulations elicited higher titers than the CPMV formulations against both S protein variants. These data further confirm potency of VNP formulations with the 826 epitope.

Finally, we assayed the avidity of the antibodies produced by our vaccine candidates. The avidity of an antibody is a measure of the overall strength of an antibody–antigen complex (Gaspar and De Gaspari, 2021). We determined the avidity of the antibodies by coating ELISA plates with the SARS-CoV-2 and Omicron (B1.1.529) RBD and testing antibody binding in the presence of 7 M urea, which encourages the detachment of low-avidity antibodies (Vogt et al., 2022). We compared the avidity of these specific antibodies at weeks 6, 16 and 50 post-immunization. Data revealed that although Qβ elicited higher antibody titers, the CPMV formulations achieved a higher avidity index compared the Qβ formulations, and 826-CPMV_100 achieved the highest value of all (Figure 5B). Overall, the implant performed similar to one bolus injection of the vaccine dose and therefore may not offer a distinct advantage for the vaccines studied.

As mentioned above, the higher titers that were produced for the Qβ vaccine candidates may be result of higher epitope density on the Qβ particle, consistent with reports demonstrating that highly ordered repetitive arrays of epitopes are effective for the induction of immune responses and breaking B-cell tolerance (Bachmann and Jennings, 2010; Mohsen et al., 2020). However, of importance is that higher avidity antibodies were generated using the CPMV platform: This may reflect the lower density of peptides on the CPMV surface, which would affect the spacing of the epitopes and thus the stability of the antibody–antigen complex (Jendroszek and Kjaergaard, 2021; Oostindie et al., 2022). In antibody engineering, it is important to know the optimal epitope spacing, the maximum spacing that can be tolerated, and how strongly avidity depends on the epitope spacing (Jendroszek and Kjaergaard, 2021; Oostindie et al., 2022).

It is also off note that we observed an increase in antibody avidity for both the SARS-CoV-2 and Omicron S-proteins as time progressed, especially for 826-CPMV_100 and 826-Qβ_implant. The virus-specific IgG avidity is expected to increase with time (Gaspar and De Gaspari, 2021) because it represents the strength of an antibody–antigen complex, and therefore the quality of the immune response (Arias-Bouda et al., 2003; Pichler et al., 2021). IgG avidity tends to be lower after the first antigenic challenge and increases over the time because later antibodies have undergone affinity maturation by somatic hypermutation, improving the response to subsequent encounters with the pathogen (Arias-Bouda et al., 2003; Pichler et al., 2021). Avidity also reveals the functionality of persistent antibodies, which influences the quality of the immune response against a pathogen.

## Conclusion

The B-cell peptide 809–826 (PSKPSKRSFIEDLLFNKV) from the SARS-CoV-2 S-protein was conjugated to CPMV and Qβ VLPs, followed by the immunization of mice using different schedules: a single dose (200 μg particles s.c.), a prime–boost regimen (100 μg particles per s.c. dose), or a polymeric implant containing 200 μg of particles. All formulations elicited sustained antibody titers for at least 50 weeks, with high avidity against the S-protein of SARS-CoV-2 Wuhan strain and a more recent Omicron subvariant. The candidates also elicited a balanced Th1/Th2 immune response, with the CPMV formulations more biased toward a Th1 response. The 826 epitope is highly conserved among SARS-CoV-2 variants of concern, explaining the high titers against the Omicron B1.1.529 strain. The B-cell epitope approach therefore achieves a more targeted antibody response that covers multiple variants of concern. Moreover, bacteriophages and plant virus nanoparticles are thermally stable, allowing the distribution of vaccines without a cold chain. The vaccine candidates can also be incorporated into a polymeric implantable scaffold by 3D printing, to achieve the sustained release of particles and maintain high antibody titers with elevated avidity at least 1 year after implantation. Our strategy offers a versatile platform for the development of new vaccines against COVID-19 and future pandemics.

## Data availability statement

The original contributions presented in the study are included in the article/Supplementary material, further inquiries can be directed to the corresponding authors.

## Ethics statement

The animal study was reviewed and approved by University of California, San Diego IACUC.

## Author contributions

JFAO: vaccine studies and immunological assays. ZZ and YX: fabrication and analysis of the implantable polymeric scaffold and contributed to writing sections of the manuscript. MDS: vaccine synthesis and characterization. KEV and XD: assistance with immunological assays and analysis. SS: initial vaccine studies. JFAO and NFS: writing of the manuscript. NFS and SC: project conceptualization, scientific oversight, funding acquisition, and team management. All authors contributed to the article and approved the submitted version.

## Funding

This work was supported in part through NIH grants R21 AI161306 and R01 CA253615 and the UC San Diego MRSEC DMR-2011924 funded by NFS. The authors would like to thank the University of California San Diego—Cellular and Molecular Medicine Electron Microscopy Core (UCSD-CMM-EM Core, RRID:SCR 022039) for equipment access and technical assistance. The UCSD-CMM-EM Core is supported in part by the National Institutes of Health Award number S10OD023527.

## Conflict of interest

NFS is a co-founder of, has equity in, and has a financial interest with Mosaic ImmunoEngineering Inc. NFS serves as Director, Board Member, and Acting Chief Scientific Officer, and paid consultant to Mosaic.

The remaining authors declare that the research was conducted in the absence of any commercial or financial relationships that could be construed as a potential conflict of interest.

## Publisher’s note

All claims expressed in this article are solely those of the authors and do not necessarily represent those of their affiliated organizations, or those of the publisher, the editors and the reviewers. Any product that may be evaluated in this article, or claim that may be made by its manufacturer, is not guaranteed or endorsed by the publisher.

## Supplementary material

The Supplementary material for this article can be found online at:

https://www.frontiersin.org/articles/10.3389/fmicb.2023.1117494/full#supplementary-material

## References

[ref1] Andrews N. Stowe J. Kirsebom F. Toffa S. Rickeard T. Gallagher E.. (2022a). Covid-19 Vaccine Effectiveness against the Omicron (B.1.1.529) Variant. N. Engl. J. Med. 386, 1532–1546. doi: 10.1056/NEJMoa2119451, PMID: 35249272 PMC8908811

[ref2] Andrews N. Tessier E. Stowe J. Gower C. Kirsebom F. Simmons R.. (2022b). Duration of protection against mild and severe disease by Covid-19 vaccines. N. Engl. J. Med. 386, 340–350. doi: 10.1056/NEJMoa2115481, PMID: 35021002 PMC8781262

[ref3] Arias-Bouda L. M. Kuijper S. Van der Werf A. Nguyen L. N. Jansen H. M. Kolk A. H. (2003). Changes in avidity and level of immunoglobulin G antibodies to Mycobacterium tuberculosis in sera of patients undergoing treatment for pulmonary tuberculosis. Clin. Diagn. Lab. Immunol. 10, 702–709. doi: 10.1128/cdli.10.4.702-709.2003, PMID: 12853408 PMC164257

[ref4] Bachmann M. F. Jennings G. T. (2010). Vaccine delivery: a matter of size, geometry, kinetics and molecular patterns. Nat. Rev. Immunol. 10, 787–796. doi: 10.1038/nri2868, PMID: 20948547

[ref5] Berger A. (2000). Th1 and Th2 responses: what are they? BMJ 321:424. doi: 10.1136/bmj.321.7258.424, PMID: 10938051 PMC27457

[ref6] Bobbala S. Gibson B. Gamble A. B. McDowell A. Hook S. (2018). Poloxamer 407-chitosan grafted thermoresponsive hydrogels achieve synchronous and sustained release of antigen and adjuvant from single-shot vaccines. Immunol. Cell Biol. 96, 656–665. doi: 10.1111/imcb.12031, PMID: 29499080

[ref7] Burki T. K. (2021). Challenges in the rollout of COVID-19 vaccines worldwide. Lancet Respir. Med. 9, e42–e43. doi: 10.1016/S2213-2600(21)00129-6, PMID: 33684355 PMC8009608

[ref8] Burki T. (2022). COVID vaccine booster doses for omicron variants. Lancet Respir. Med. 10:936. doi: 10.1016/s2213-2600(22)00361-7, PMID: 36063833 PMC9439639

[ref9] Caccavo D. Cascone S. Lamberti G. Barba A. A. (2015). Controlled drug release from hydrogel-based matrices: experiments and modeling. Int. J. Pharm. 486, 144–152. doi: 10.1016/j.ijpharm.2015.03.054, PMID: 25827589

[ref10] Chalkias S. Harper C. Vrbicky K. Walsh S. R. Essink B. Brosz A.. (2022). A bivalent omicron-containing booster vaccine against Covid-19. N. Engl. J. Med. 387, 1279–1291. doi: 10.1056/NEJMoa2208343, PMID: 36112399 PMC9511634

[ref11] Chariou P. L. Ortega-Rivera O. A. Steinmetz N. F. (2020). Nanocarriers for the delivery of medical, veterinary, and agricultural active ingredients. ACS Nano 14, 2678–2701. doi: 10.1021/acsnano.0c00173, PMID: 32125825 PMC8085836

[ref12] Chung Y. H. Beiss V. Fiering S. N. Steinmetz N. F. (2020). COVID-19 vaccine frontrunners and their nanotechnology design. ACS Nano 14, 12522–12537. doi: 10.1021/acsnano.0c07197, PMID: 33034449

[ref13] Chung N. H. Chen Y. C. Yang S. J. Lin Y. C. Dou H. Y. Hui-Ching Wang L.. (2022). Induction of Th1 and Th2 in the protection against SARS-CoV-2 through mucosal delivery of an adenovirus vaccine expressing an engineered spike protein. Vaccine 40, 574–586. doi: 10.1016/j.vaccine.2021.12.024, PMID: 34952759 PMC8677488

[ref14] Collier A. Y. Yu J. McMahan K. Liu J. Chandrashekar A. Maron J. S.. (2021). Differential kinetics of immune responses elicited by Covid-19 vaccines. N. Engl. J. Med. 385, 2010–2012. doi: 10.1056/NEJMc2115596, PMID: 34648703 PMC8531985

[ref15] Dejnirattisai W. Zhou D. Supasa P. Liu C. Mentzer A. J. Ginn H. M.. (2021). Screaton: Antibody evasion by the P.1 strain of SARS-CoV-2. Cells 184, 2939–2954.e9. doi: 10.1016/j.cell.2021.03.055, PMID: 33852911 PMC8008340

[ref16] Fairbanks B. D. Schwartz M. P. Bowman C. N. Anseth K. S. (2009). Photoinitiated polymerization of PEG-diacrylate with lithium phenyl-2,4,6-trimethylbenzoylphosphinate: polymerization rate and cytocompatibility. Biomaterials 30, 6702–6707. doi: 10.1016/j.biomaterials.2009.08.055, PMID: 19783300 PMC2896013

[ref17] Forgacs D. Jang H. Abreu R. B. Hanley H. B. Gattiker J. L. Jefferson A. M.. (2021). SARS-CoV-2 mRNA vaccines elicit different responses in immunologically naive and pre-immune humans. Front. Immunol. 12:728021. doi: 10.3389/fimmu.2021.728021, PMID: 34646267 PMC8502960

[ref18] Gaspar E. B. De Gaspari E. (2021). Avidity assay to test functionality of anti-SARS-Cov-2 antibodies. Vaccine 39, 1473–1475. doi: 10.1016/j.vaccine.2021.02.003, PMID: 33581919 PMC7857056

[ref19] Goel R. R. Painter M. M. Apostolidis S. A. Mathew D. Meng W. Rosenfeld A. M.. (2021). mRNA vaccines induce durable immune memory to SARS-CoV-2 and variants of concern. Science 374:abm0829. doi: 10.1126/science.abm0829 34648302 PMC9284784

[ref20] Hajnik R. L. Plante J. A. Liang Y. Alameh M. G. Tang J. Bonam S. R.. (2022). Dual spike and nucleocapsid mRNA vaccination confer protection against SARS-CoV-2 Omicron and Delta variants in preclinical models. Sci. Transl. Med. 14:eabq1945. doi: 10.1126/scitranslmed.abq1945, PMID: 36103514 PMC9926941

[ref21] Hou Y. Liu R. Hong X. Zhang Y. Bai S. Luo X.. (2021). Engineering a sustained release vaccine with a pathogen-mimicking manner for robust and durable immune responses. J. Control. Release 333, 162–175. doi: 10.1016/j.jconrel.2021.03.037, PMID: 33794269

[ref22] Huang Q. Ji K. Tian S. Wang F. Huang B. Tong Z.. (2021). A single-dose mRNA vaccine provides a long-term protection for hACE2 transgenic mice from SARS-CoV-2. Nat. Commun. 12:776. doi: 10.1038/s41467-021-21037-2, PMID: 33536425 PMC7858593

[ref23] Hui D. S. (2022). Hybrid immunity and strategies for COVID-19 vaccination. Lancet Infect. Dis. 23, 2–3. doi: 10.1016/s1473-3099(22)00640-5, PMID: 36152670 PMC9761779

[ref24] Huynh N. T. Hesketh E. L. Saxena P. Meshcheriakova Y. Ku Y. C. Hoang L. T.. (2016). Crystal structure and proteomics analysis of empty virus-like particles of cowpea mosaic virus. Structure 24, 567–575. doi: 10.1016/j.str.2016.02.011, PMID: 27021160 PMC4828962

[ref25] Jalkanen P. Kolehmainen P. Hakkinen H. K. Huttunen M. Tahtinen P. A. Lundberg R.. (2021). COVID-19 mRNA vaccine induced antibody responses against three SARS-CoV-2 variants. Nat. Commun. 12:3991. doi: 10.1038/s41467-021-24285-4, PMID: 34183681 PMC8239026

[ref26] Jendroszek A. Kjaergaard M. (2021). Nanoscale spatial dependence of avidity in an IgG1 antibody. Sci. Rep. 11:12663. doi: 10.1038/s41598-021-92280-2, PMID: 34135438 PMC8209022

[ref27] Kowalzik F. Schreiner D. Jensen C. Teschner D. Gehring S. Zepp F. (2021). mRNA-based vaccines. Vaccines (Basel) 9:390. doi: 10.3390/vaccines9040390, PMID: 33921028 PMC8103517

[ref28] Lai C. C. Shih T. P. Ko W. C. Tang H. J. Hsueh P. R. (2020). Severe acute respiratory syndrome coronavirus 2 (SARS-CoV-2) and coronavirus disease-2019 (COVID-19): The epidemic and the challenges. Int. J. Antimicrob. Agents 55:105924. doi: 10.1016/j.ijantimicag.2020.105924, PMID: 32081636 PMC7127800

[ref29] Lebel M. E. Chartrand K. Leclerc D. Lamarre A. (2015). Plant viruses as nanoparticle-based vaccines and adjuvants. Vaccines (Basel) 3, 620–637. doi: 10.3390/vaccines3030620, PMID: 26350598 PMC4586470

[ref30] Lee W. S. Wheatley A. K. Kent S. J. DeKosky B. J. (2020). Antibody-dependent enhancement and SARS-CoV-2 vaccines and therapies. Nat. Microbiol. 5, 1185–1191. doi: 10.1038/s41564-020-00789-5 32908214 PMC12103240

[ref31] Lee A. L. Z. Yang C. Gao S. Hedrick J. L. Yang Y. Y. (2019). Subcutaneous vaccination using injectable biodegradable hydrogels for long-term immune response. Nanomedicine 21:102056. doi: 10.1016/j.nano.2019.102056, PMID: 31336176

[ref32] Li X. (2022). Omicron: call for updated vaccines. J. Med. Virol. 94, 1261–1263. doi: 10.1002/jmv.27530, PMID: 34927258

[ref33] Li Y. Lai D. Y. Zhang H. N. Jiang H. W. Tian X. Ma M. L.. (2020). Linear epitopes of SARS-CoV-2 spike protein elicit neutralizing antibodies in COVID-19 patients. Cell. Mol. Immunol. 17, 1095–1097. doi: 10.1038/s41423-020-00523-5, PMID: 32895485 PMC7475724

[ref34] Madi M. Mioulet V. King D. P. Lomonossoff G. P. Montague N. P. (2015). Development of a non-infectious encapsidated positive control RNA for molecular assays to detect foot-and-mouth disease virus. J. Virol. Methods 220, 27–34. doi: 10.1016/j.jviromet.2015.04.002, PMID: 25864934 PMC4451496

[ref35] Mathieu E. Ritchie H. Ortiz-Ospina E. Roser M. Hasell J. Appel C.. (2021). A global database of COVID-19 vaccinations. Nat. Hum. Behav. 5, 947–953. doi: 10.1038/s41562-021-01122-8 33972767

[ref36] Mohsen M. O. Augusto G. Bachmann M. F. (2020). The 3Ds in virus-like particle based-vaccines: "design, delivery and dynamics". Immunol. Rev. 296, 155–168. doi: 10.1111/imr.12863, PMID: 32472710 PMC7496916

[ref37] Montague N. P. Thuenemann E. C. Saxena P. Saunders K. Lenzi P. Lomonossoff G. P. (2011). Recent advances of cowpea mosaic virus-based particle technology. Hum. Vaccin. 7, 383–390. doi: 10.4161/hv.7.3.14989, PMID: 21368585

[ref38] Nagpal D. Nagpal S. Kaushik D. Kathuria H. (2022). Current clinical status of new COVID-19 vaccines and immunotherapy. Environ. Sci. Pollut. Res. Int. 29, 70772–70807. doi: 10.1007/s11356-022-22661-1, PMID: 36063274 PMC9442597

[ref39] Nkanga C. I. Ortega-Rivera O. A. Shin M. D. Moreno-Gonzalez M. A. Steinmetz N. F. (2022). Injectable slow-release hydrogel formulation of a plant virus-based COVID-19 vaccine candidate. Biomacromolecules 23, 1812–1825. doi: 10.1021/acs.biomac.2c00112, PMID: 35344365

[ref40] Oostindie S. C. Lazar G. A. Schuurman J. Parren P. (2022). Avidity in antibody effector functions and biotherapeutic drug design. Nat. Rev. Drug Discov. 21, 715–735. doi: 10.1038/s41573-022-00501-8, PMID: 35790857 PMC9255845

[ref41] Ortega-Rivera O. A. Pokorski J. K. Steinmetz N. F. (2021). A single-dose, implant-based, trivalent virus-like particle vaccine against "cholesterol checkpoint" proteins. Adv Ther (Weinh) 4:2100014. doi: 10.1002/adtp.202100014, PMID: 34541299 PMC8447230

[ref42] Ortega-Rivera O. A. Shin M. D. Chen A. Beiss V. Moreno-Gonzalez M. A. Lopez-Ramirez M. A.. (2021). Trivalent subunit vaccine candidates for COVID-19 and their delivery devices. J. Am. Chem. Soc. 143, 14748–14765. doi: 10.1021/jacs.1c06600, PMID: 34490778

[ref43] Patel R. Kaki M. Potluri V. S. Kahar P. Khanna D. (2022). A comprehensive review of SARS-CoV-2 vaccines: Pfizer, Moderna & Johnson & Johnson. Hum. Vaccin. Immunother. 18:2002083. doi: 10.1080/21645515.2021.2002083, PMID: 35130825 PMC8862159

[ref44] Pichler D. Baumgartner M. Kimpel J. Rossler A. Riepler L. Bates K.. (2021). Marked increase in avidity of SARS-CoV-2 antibodies 7-8 months after infection is not diminished in old age. J. Infect. Dis. 224, 764–770. doi: 10.1093/infdis/jiab300, PMID: 34086960 PMC8195195

[ref45] Qian Wang T. L. Tang L. Johnson J. E. Finn M. G. (2002). Icosahedral virus particles as addressable nanoscale building blocks. Angew. Chem. Int. Ed. 41, 459–462. doi: 10.1002/1521-3773(20020201)41:3<459::AID-ANIE459>3.0.CO;2-O, PMID: 12491378

[ref46] Rosenthal K. S. Zimmerman D. H. (2006). Vaccines: all things considered. Clin. Vaccine Immunol. 13, 821–829. doi: 10.1128/CVI.00152-06, PMID: 16893980 PMC1539119

[ref47] Sah P. Vilches T. N. Moghadas S. M. Fitzpatrick M. C. Singer B. H. Hotez P. J.. (2021). Accelerated vaccine rollout is imperative to mitigate highly transmissible COVID-19 variants. EClinicalMedicine 35:100865. doi: 10.1016/j.eclinm.2021.100865, PMID: 33937735 PMC8072134

[ref48] Shin M. D. Hochberg J. D. Pokorski J. K. Steinmetz N. F. (2021). Bioconjugation of active ingredients to plant viral nanoparticles is enhanced by preincubation with a pluronic F127 polymer scaffold. ACS Appl. Mater. Interfaces 13, 59618–59632. doi: 10.1021/acsami.1c13183, PMID: 34890195 PMC11729460

[ref49] Shin M. D. Shukla S. Chung Y. H. Beiss V. Chan S. K. Ortega-Rivera O. A.. (2020). COVID-19 vaccine development and a potential nanomaterial path forward. Nat. Nanotechnol. 15, 646–655. doi: 10.1038/s41565-020-0737-y, PMID: 32669664

[ref50] Smith K. M. Pottage L. Thomas E. R. Leishman A. J. Doig T. N. Xu D.. (2000). Th1 and Th2 CD4+ T cells provide help for B cell clonal expansion and antibody synthesis in a similar manner in vivo. J. Immunol. 165, 3136–3144. doi: 10.4049/jimmunol.165.6.3136, PMID: 10975827

[ref51] Soman P. Chung P. H. Zhang A. P. Chen S. (2013). Digital microfabrication of user-defined 3D microstructures in cell-laden hydrogels. Biotechnol. Bioeng. 110, 3038–3047. doi: 10.1002/bit.24957, PMID: 23686741 PMC3784638

[ref52] Sun Z. Song C. Wang C. Hu Y. Wu J. (2020). Hydrogel-based controlled drug delivery for cancer treatment: a review. Mol. Pharm. 17, 373–391. doi: 10.1021/acs.molpharmaceut.9b01020, PMID: 31877054

[ref53] Szabo G. T. Mahiny A. J. Vlatkovic I. (2022). COVID-19 mRNA vaccines: platforms and current developments. Mol. Ther. 30, 1850–1868. doi: 10.1016/j.ymthe.2022.02.016, PMID: 35189345 PMC8856755

[ref54] Tang P. Hasan M. R. Chemaitelly H. Yassine H. M. Benslimane F. M. Al Khatib H. A.. (2021). BNT162b2 and mRNA-1273 COVID-19 vaccine effectiveness against the SARS-CoV-2 Delta variant in Qatar. Nat. Med. 27, 2136–2143. doi: 10.1038/s41591-021-01583-4, PMID: 34728831

[ref55] Vogt A. S. Jorg L. Martina B. Krenger P. S. Chang X. Zeltins A.. (2022). Virus-like particles are efficient tools for boosting mRNA-induced antibodies. Front. Immunol. 13:864718. doi: 10.3389/fimmu.2022.864718, PMID: 35784292 PMC9245429

[ref56] Wang Q. Zhang L. Kuwahara K. Li L. Liu Z. Li T.. (2016). Immunodominant SARS coronavirus epitopes in humans elicited both enhancing and neutralizing effects on infection in non-human primates. ACS Infect Dis 2, 361–376. doi: 10.1021/acsinfecdis.6b00006, PMID: 27627203 PMC7075522

[ref57] Watson O. J. Barnsley G. Toor J. Hogan A. B. Winskill P. Ghani A. C. (2022). Global impact of the first year of COVID-19 vaccination: a mathematical modelling study. Lancet Infect. Dis. 22, 1293–1302. doi: 10.1016/s1473-3099(22)00320-6, PMID: 35753318 PMC9225255

[ref58] Weidenbacher P. A. Waltari E. de Los Rios I. Kobara B. N. Bell M. K. Morris Y. C.. (2022). Converting non-neutralizing SARS-CoV-2 antibodies into broad-spectrum inhibitors. Nat. Chem. Biol. 18, 1270–1276. doi: 10.1038/s41589-022-01140-1, PMID: 36076082 PMC9596371

[ref59] Wellink J. (1998). Comovirus isolation and RNA extraction. Methods Mol. Biol. 81, 205–209. doi: 10.1385/0-89603-385-6:205, PMID: 9760508

[ref60] Wu Y. Wang F. Shen C. Peng W. Li D. Zhao C.. (2020). A noncompeting pair of human neutralizing antibodies block COVID-19 virus binding to its receptor ACE2. Science 368, 1274–1278. doi: 10.1126/science.abc2241, PMID: 32404477 PMC7223722

[ref61] Zanella I. Degli Antoni M. Marchese V. Castelli F. Quiros-Roldan E. (2022). Non-neutralizing antibodies: Deleterious or propitious during SARS-CoV-2 infection? Int. Immunopharmacol. 110:108943. doi: 10.1016/j.intimp.2022.108943, PMID: 35753123 PMC9189100

[ref62] Zhou D. Dejnirattisai W. Supasa P. Liu C. Mentzer A. J. Ginn H. M.. (2021). Screaton: Evidence of escape of SARS-CoV-2 variant B.1.351 from natural and vaccine-induced sera. Cells 184, 2348–2361.e6. doi: 10.1016/j.cell.2021.02.037, PMID: 33730597 PMC7901269

