# Supplementary material for: COVID-19 vaccines based on viral nanoparticles displaying a conserved B-cell epitope show potent immunogenicity and a long-lasting antibody response
Source: Front Microbiol. 2023 Apr 20;14:1117494. doi: 10.3389/fmicb.2023.1117494 (PMC10157238; doi:10.3389/fmicb.2023.1117494)
Supplement: Supplementary file 1 [file Data_Sheet_1.PDF]

SUPPORTING INFORMATION

COVID-19 vaccines based on viral nanoparticles displaying a conserved B-cell epitope show potent immunogenicity and a long-lasting antibody response

Jessica Fernanda Affonso de Oliveira[1], Zhongchao Zhao[1,2,3], Yi Xiang[1], Matthew D. Shin[1], Kathleen Elizabeth Villaseñor[4], Xinyi Deng[1], Sourabh Shukla[1], Shaochen Chen[1,2,5,7*], and Nicole F. Steinmetz [1,2,3,5,6,7, 8*]

[1]Department of NanoEngineering, University of California San Diego, 9500 Gilman Dr., La Jolla, CA 92093, USA
[2]Center for Nano-ImmunoEngineering, University of California San Diego, 9500 Gilman Dr., La Jolla, CA 92093, USA.
[3]Moores Cancer Center, University of California San Diego, 9500 Gilman Dr., La Jolla, CA 92093, USA.
[4]Department of Bioengineering, University of California Los Angeles, 405 Hilgard Avenue, Los Angeles, CA, 90095
[5]Department of Bioengineering, University of California San Diego, 9500 Gilman Dr., La Jolla, CA 92093, USA.
[6]Department of Radiology, University of California San Diego, 9500 Gilman Dr., La Jolla, CA 92093, USA.
[7]Institute for Materials Discovery and Design, University of California San Diego, 9500 Gilman Dr., La Jolla, CA 92093, USA.
[8]Center for Engineering in Cancer, University of California San Diego, 9500 Gilman Dr., La Jolla, CA 92093 USA

*** Correspondence:**
Corresponding Author
chen168@eng.ucsd.edu; nsteinmetz@ucsd.edu

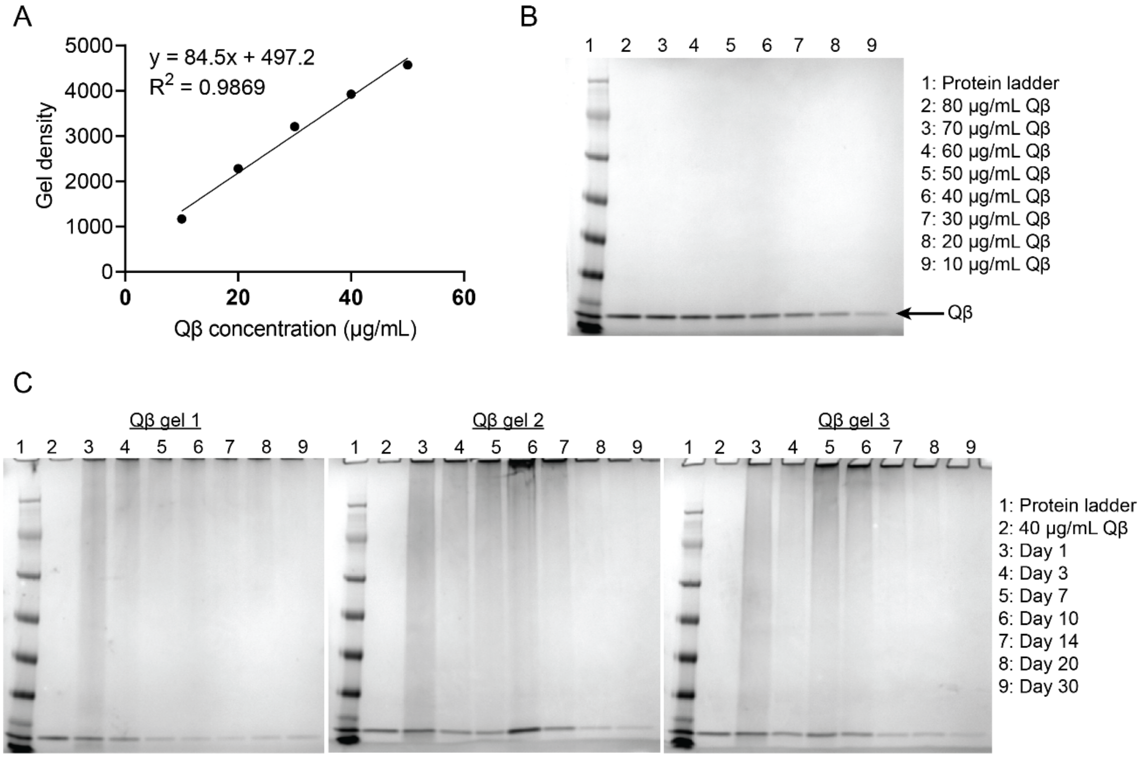

Figure S1. *In vitro* release of Qβ particles from implants. (A) Qβ standard curve based on (B) SDS-PAGE and densitometric analysis with different Qβ loads. (C) SDS-PAGE analysis of Qβ particles released from three implants at different time points.

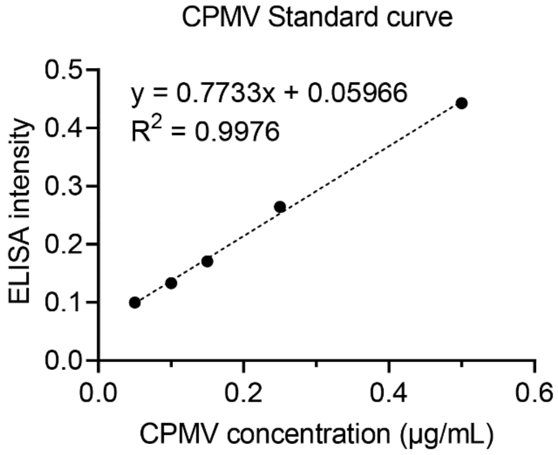

Figure S2. CPMV standard curve based on the analysis of different concentrations of CPMV by ELISA.

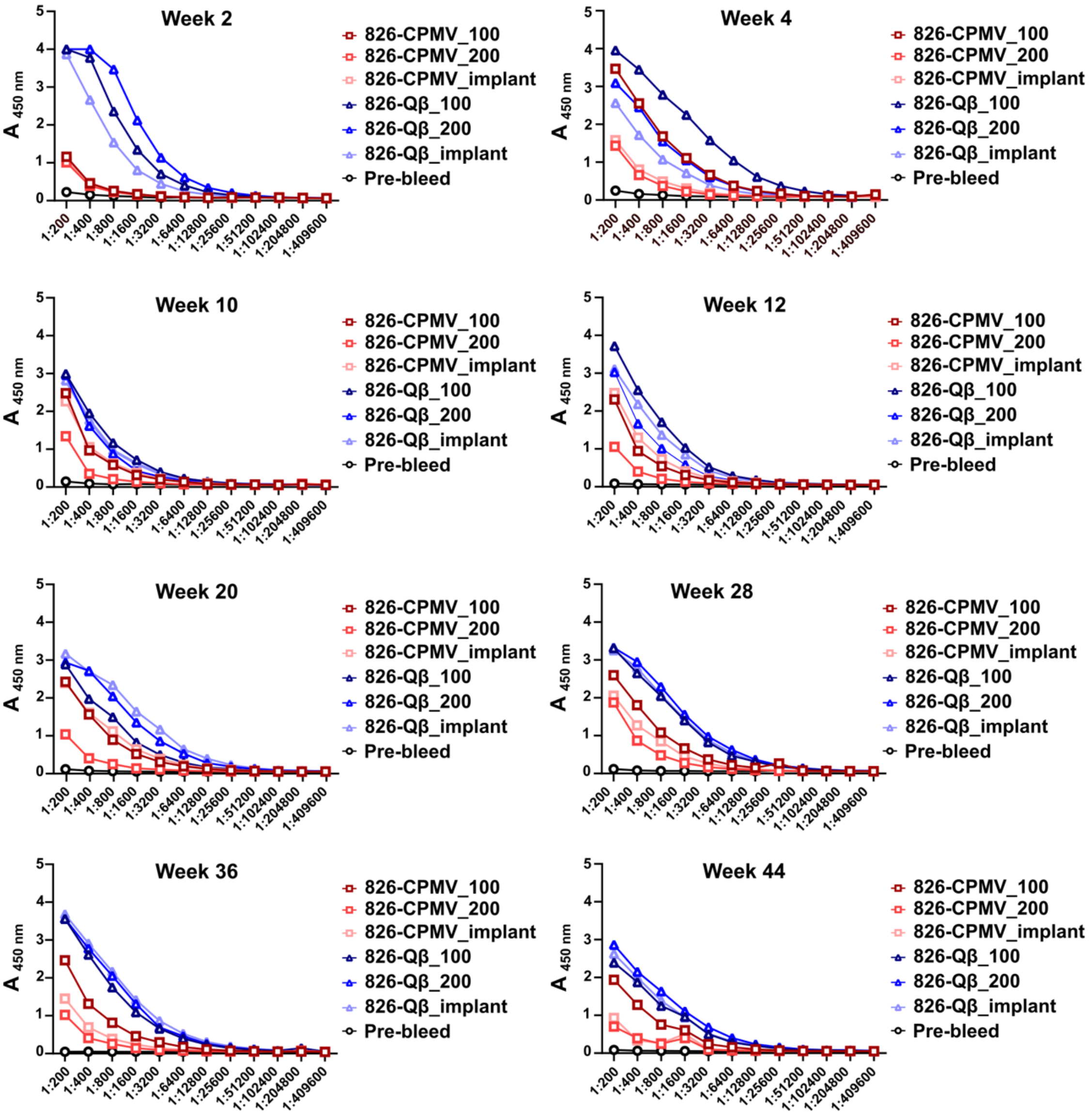

Figure S3. Immunogenicity of CPMV/Qβ vaccine candidates. Mice received (1) two doses of 100 μg particles (826-CPMV_100 or 826-Qβ_100), (2) one dose of 200 μg particles (826-CPMV_200 or 826-Qβ_200) or (3) the slow-release implant (826-CPMV_implant or 826-Qβ_implant). Antibody titers against the 826 epitope were evaluated at different time points post-immunization.

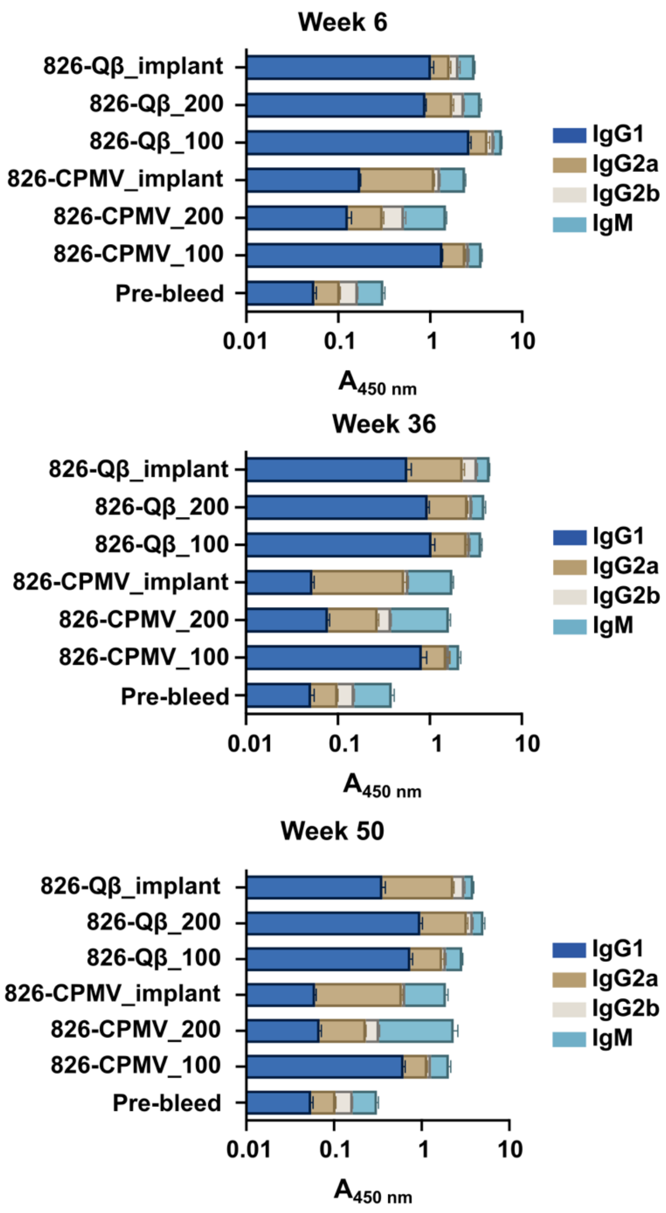

Figure S4. Isotype profile (IgG and IgM) and IgG subclass profile (IgG1, IgG2a and IgG2b) of sera isolated from mice immunized with different vaccine candidate formulations, determined at weeks 6, 36 and 50.

Table S1. Two-way ANOVA for Figure 3 using pairwise multiple comparison followed by Tukey's multiple comparison test was used to compare between groups. Asterisks indicate significant differences between groups (*p < 0.05; **p < 0.01, **** p <0.0001).

Tukey's multiple comparisons test - Week 6 titers, Statistically significant difference and P value

	826-CPMV_100	826-CPMV_200	826-CPMV_implant	826-Qβ_100	826-Qβ_200	826-Qβ_implant	Pre-bleed
**826-CPMV_100**	N/A	*; P = 0.04	ns; P = 0.9566	ns; P = 0.9051	ns; P = 0.8482	ns; P = 0.6856	**; P = 0.0094
**826-CPMV_200**		N/A	ns; P = 0.3391	**; P = 0.0011	ns; P = 0.5378	ns; P = 0.7258	ns; P = 0.9987
**826-CPMV_implant**			N/A	ns; P = 0.3401	ns; P = >0.9999	ns; P = 0.9961	ns; P = 0.1269
**826-Qβ_100**				N/A	ns; P = 0.1887	ns; P = 0.1	***; P = 0.0002
**826-Qβ_200**					N/A	ns; P = >0.9999	ns; P = 0.2452
**826-Qβ_implant**						N/A	ns; P = 0.4011
**Pre-bleed**							N/A

Tukey's multiple comparisons test - Week 16 titers, Statistically significant difference and P value

	826-CPMV_100	826-CPMV_200	826-CPMV_implant	826-Qβ_100	826-Qβ_200	826-Qβ_implant	Pre-bleed
**826-CPMV_100**	N/A	ns; P = 0.8979	ns; P = 0.9991	ns; P = 0.1999	ns; P = 0.7828	ns; P = 0.2622	ns; P = 0.426
**826-CPMV_200**		N/A	ns; P = 0.6484	**; P = 0.0098	ns; P = 0.1381	*; P = 0.0149	ns; P = 0.9827
**826-CPMV_implant**			N/A	ns; P = 0.445	ns; P = 0.9605	ns; P = 0.537	ns; P = 0.1881
**826-Qβ_100**				N/A	ns; P = 0.9509	ns; P = >0.9999	***; P = 0.0007
**826-Qβ_200**					N/A	ns; P = 0.9767	*; P = 0.0172
**826-Qβ_implant**						N/A	**; P = 0.0011
**Pre-bleed**							N/A

Tukey's multiple comparisons test - Week 50 titers, Statistically significant difference and P value

	826-CPMV_100	826-CPMV_200	826-CPMV_implant	826-Qβ_100	826-Qβ_200	826-Qβ_implant	Pre-bleed
**826-CPMV_100**	N/A	ns; P = 0.6716	ns; P = 0.7138	ns; P = 0.9955	ns; P = 0.2769	ns; P = 0.4847	ns; P = 0.2377
**826-CPMV_200**		N/A	ns; P = >0.9999	ns; P = 0.2848	**; P = 0.0043	*; P = 0.0131	ns; P = 0.9902
**826-CPMV_implant**			N/A	ns; P = 0.3198	**; P = 0.0054	*; P = 0.016	ns; P = 0.9844
**826-Qβ_100**				N/A	ns; P = 0.6616	ns; P = 0.8613	ns; P = 0.0587
**826-Qβ_200**					N/A	ns; P = 0.9998	***; P = 0.0004
**826-Qβ_implant**						N/A	**; P = 0.0013
**Pre-bleed**							N/A

Table S2. Two-way ANOVA for Figure 4A using pairwise multiple comparison followed by Tukey's multiple comparison test was used to compare between groups. Asterisks indicate significant differences between groups (*p < 0.05; **p < 0.01, **** p <0.0001).

Tukey's multiple comparisons test - Week 2, Statistically significant difference and P value

	826-CPMV_100	826-CPMV_200	826-CPMV_implant	826-Qβ_100	826-Qβ_200	826-Qβ_implant	mRNA-RBD
826-CPMV_100	N/A	ns; P = 0.1792	ns; P = 0.6815	*; P = 0.038	ns; P = 0.106	ns; P = 0.8219	**; P = 0.0073
826-CPMV_200		N/A	ns; P = 0.914	***; P = 0.0002	***; P = 0.0005	*; P = 0.0156	***; P = 0.0003
826-CPMV_implant			N/A	**; P = 0.0015	**; P = 0.0047	ns; P = 0.1196	**; P = 0.0022
826-Qβ_100				N/A	ns; P = 0.995	ns; P = 0.3481	ns; P = 0.2321
826-Qβ_200					N/A	ns; P = 0.6431	ns; P = 0.0733
826-Qβ_implant						N/A	*; P = 0.0214
mRNA-RBD							N/A

Tukey's multiple comparisons test - Week 6, Statistically significant difference and P value

	826-CPMV_100	826-CPMV_200	826-CPMV_implant	826-Qβ_100	826-Qβ_200	826-Qβ_implant	mRNA-RBD
826-CPMV_100	N/A	ns; P = 0.1792	ns; P = 0.6815	*; P = 0.038	ns; P = 0.106	ns; P = 0.8219	**; P = 0.0073
826-CPMV_200		N/A	ns; P = 0.914	***; P = 0.0002	***; P = 0.0005	*; P = 0.0156	***; P = 0.0003
826-CPMV_implant			N/A	**; P = 0.0015	**; P = 0.0047	ns; P = 0.1196	**; P = 0.0022
826-Qβ_100				N/A	ns; P = 0.995	ns; P = 0.3481	ns; P = 0.2321
826-Qβ_200					N/A	ns; P = 0.6431	ns; P = 0.0733
826-Qβ_implant						N/A	*; P = 0.0214
mRNA-RBD							N/A

Tukey's multiple comparisons test - Week 20, Statistically significant difference and P value

	826-CPMV_100	826-CPMV_200	826-CPMV_implant	826-Qβ_100	826-Qβ_200	826-Qβ_implant	mRNA-RBD
826-CPMV_100	N/A	ns; P = 0.1792	ns; P = 0.6815	*; P = 0.038	ns; P = 0.106	ns; P = 0.8219	**; P = 0.0073
826-CPMV_200		N/A	ns; P = 0.914	***; P = 0.0002	***; P = 0.0005	*; P = 0.0156	***; P = 0.0003
826-CPMV_implant			N/A	**; P = 0.0015	**; P = 0.0047	ns; P = 0.1196	**; P = 0.0022
826-Qβ_100				N/A	ns; P = 0.995	ns; P = 0.3481	ns; P = 0.2321
826-Qβ_200					N/A	ns; P = 0.6431	ns; P = 0.0733
826-Qβ_implant						N/A	*; P = 0.0214
mRNA-RBD							N/A

Tukey's multiple comparisons test - Week 28, Statistically significant difference and P value

	826-CPMV_100	826-CPMV_200	826-CPMV_implant	826-Qβ_100	826-Qβ_200	826-Qβ_implant	mRNA-RBD
826-CPMV_100	N/A	ns; P = 0.1792	ns; P = 0.6815	*; P = 0.038	ns; P = 0.106	ns; P = 0.8219	**; P = 0.0073
826-CPMV_200		N/A	ns; P = 0.914	***; P = 0.0002	***; P = 0.0005	*; P = 0.0156	***; P = 0.0003
826-CPMV_implant			N/A	**; P = 0.0015	**; P = 0.0047	ns; P = 0.1196	**; P = 0.0022
826-Qβ_100				N/A	ns; P = 0.995	ns; P = 0.3481	ns; P = 0.2321
826-Qβ_200					N/A	ns; P = 0.6431	ns; P = 0.0733
826-Qβ_implant						N/A	*; P = 0.0214
mRNA-RBD							N/A

Tukey's multiple comparisons test - Week 50, Statistically significant difference and P value

	826-CPMV_100	826-CPMV_200	826-CPMV_implant	826-Qβ_100	826-Qβ_200	826-Qβ_implant	mRNA-RBD
826-CPMV_100	N/A	ns; P = 0.1792	ns; P = 0.6815	*; P = 0.038	ns; P = 0.106	ns; P = 0.8219	**; P = 0.0073
826-CPMV_200		N/A	ns; P = 0.914	***; P = 0.0002	***; P = 0.0005	*; P = 0.0156	***; P = 0.0003
826-CPMV_implant			N/A	**; P = 0.0015	**; P = 0.0047	ns; P = 0.1196	**; P = 0.0022
826-Qβ_100				N/A	ns; P = 0.995	ns; P = 0.3481	ns; P = 0.2321
826-Qβ_200					N/A	ns; P = 0.6431	ns; P = 0.0733
826-Qβ_implant						N/A	*; P = 0.0214
mRNA-RBD							N/A

Table S3. Two-way ANOVA for Figure 4B using pairwise multiple comparison followed by Tukey's multiple comparison test was used to compare between groups. Asterisks indicate significant differences between groups (*p < 0.05; **p < 0.01, **** p <0.0001).

Tukey's multiple comparisons test - Week 6, Statistically significant difference and P value

	826-CPMV_100	826-CPMV_200	826-CPMV_implant	826-Qβ_100	826-Qβ_200	826-Qβ_implant
826-CPMV_100	N/A	ns; P = 0.5484	****; P = <0.0001	ns; P = 0.9995	ns; P = 0.851	ns; P = 0.201
826-CPMV_200		N/A	****; P = <0.0001	ns; P = 0.7294	ns; P = 0.9928	ns; P = 0.973
826-CPMV_implant			N/A	****; P = <0.0001	****; P = <0.0001	***; P = 0.0002
826-Qβ_100				N/A	ns; P = 0.9542	ns; P = 0.3203
826-Qβ_200					N/A	ns; P = 0.7836
826-Qβ_implant						N/A

Tukey's multiple comparisons test - Week 20, Statistically significant difference and P value

	826-CPMV_100	826-CPMV_200	826-CPMV_implant	826-Qβ_100	826-Qβ_200	826-Qβ_implant
826-CPMV_100	N/A	ns; P = 0.5484	****; P = <0.0001	ns; P = 0.9995	ns; P = 0.851	ns; P = 0.201
826-CPMV_200		N/A	****; P = <0.0001	ns; P = 0.7294	ns; P = 0.9928	ns; P = 0.973
826-CPMV_implant			N/A	****; P = <0.0001	****; P = <0.0001	***; P = 0.0002
826-Qβ_100				N/A	ns; P = 0.9542	ns; P = 0.3203
826-Qβ_200					N/A	ns; P = 0.7836
826-Qβ_implant						N/A

Tukey's multiple comparisons test - Week 36, Statistically significant difference and P value

	826-CPMV_100	826-CPMV_200	826-CPMV_implant	826-Qβ_100	826-Qβ_200	826-Qβ_implant
826-CPMV_100	N/A	ns; P = 0.5484	****; P = <0.0001	ns; P = 0.9995	ns; P = 0.851	ns; P = 0.201
826-CPMV_200		N/A	****; P = <0.0001	ns; P = 0.7294	ns; P = 0.9928	ns; P = 0.973
826-CPMV_implant			N/A	****; P = <0.0001	****; P = <0.0001	***; P = 0.0002
826-Qβ_100				N/A	ns; P = 0.9542	ns; P = 0.3203
826-Qβ_200					N/A	ns; P = 0.7836
826-Qβ_implant						N/A

Tukey's multiple comparisons test - Week 50, Statistically significant difference and P value

	826-CPMV_100	826-CPMV_200	826-CPMV_implant	826-Qβ_100	826-Qβ_200	826-Qβ_implant
826-CPMV_100	N/A	ns; P = 0.5484	****; P = <0.0001	ns; P = 0.9995	ns; P = 0.851	ns; P = 0.201
826-CPMV_200		N/A	****; P = <0.0001	ns; P = 0.7294	ns; P = 0.9928	ns; P = 0.973
826-CPMV_implant			N/A	****; P = <0.0001	****; P = <0.0001	***; P = 0.0002
826-Qβ_100				N/A	ns; P = 0.9542	ns; P = 0.3203
826-Qβ_200					N/A	ns; P = 0.7836
826-Qβ_implant						N/A

Table S4. One-way analysis of variance (ANOVA) for Figure 5A followed by Tukey's multiple comparison test was used to compare between groups. Asterisks in indicate significant differences between groups (*p < 0.05; **p < 0.01, **** p <0.0001).

IgG binding to SARS-CoV-2 S protein

Tukey's multiple comparisons test (one-way ANOVA) - Week 16, Statistically significant difference and P value

	826-CPMV_100	826-CPMV_200	826-CPMV_implant	826-Qβ_100	826-Qβ_200	826-Qβ_implant	Pre-bleed
826-CPMV_100	N/A	**; P = 0.0064	ns; P = 0.0516	****; P = <0.0001	****; P = <0.0001	*; P = 0.0108	****; P = <0.0001
826-CPMV_200		N/A	ns; P = 0.9993	****; P = <0.0001	****; P = <0.0001	ns; P = >0.9999	****; P = <0.0001
826-CPMV_implant			N/A	****; P = <0.0001	****; P = <0.0001	ns; P = >0.9999	****; P = <0.0001
826-Qβ_100				N/A	ns; P = >0.9999	****; P = <0.0001	****; P = <0.0001
826-Qβ_200					N/A	****; P = <0.0001	****; P = <0.0001
826-Qβ_implant						N/A	****; P = <0.0001
Pre-bleed							N/A

Tukey's multiple comparisons test (one-way ANOVA) - Week 50, Statistically significant difference and P value

	826-CPMV_100	826-CPMV_200	826-CPMV_implant	826-Qβ_100	826-Qβ_200	826-Qβ_implant	Pre-bleed
826-CPMV_100	N/A	ns; P = >0.9999	ns; P = 0.088	ns; P = 0.7015	****; P = <0.0001	ns; P = 0.9551	****; P = <0.0001
826-CPMV_200		N/A	ns; P = 0.0986	ns; P = 0.6676	****; P = <0.0001	ns; P = 0.9423	****; P = <0.0001
826-CPMV_implant			N/A	***; P = 0.0007	****; P = <0.0001	**; P = 0.003	***; P = 0.0007
826-Qβ_100				N/A	****; P = <0.0001	ns; P = >0.9999	****; P = <0.0001
826-Qβ_200					N/A	****; P = <0.0001	****; P = <0.0001
826-Qβ_implant						N/A	ns; P = 0.0793
Pre-bleed							N/A

Tukey's multiple comparisons test (one-way ANOVA) - Comparison between Weeks 16 and 50, Statistically significant difference and P value

	826-CPMV_100_W50	826-CPMV_200_W50	826-CPMV_implant_W50	826-Qβ_100_W50	826-Qβ_200_W50	826-Qβ_implant_W50
826-CPMV_100_W6	****; P = <0.0001	****; P = <0.0001	****; P = <0.0001	****; P = <0.0001	**; P = 0.01	****; P = <0.0001
826-CPMV_200_W6	***; P = 0.0006	***; P = 0.0006	****; P = <0.0001	ns; P = 0.0842	****; P = <0.0001	*; P = 0.0228
826-CPMV_implant_W6	****; P = <0.0001	****; P = <0.0001	****; P = <0.0001	*; P = 0.0112	****; P = <0.0001	**; P = 0.0026
826-Qβ_100_W6	****; P = <0.0001	****; P = <0.0001	****; P = <0.0001	****; P = <0.0001	**; P = 0.0093	****; P = <0.0001
826-Qβ_200_W6	****; P = <0.0001	****; P = <0.0001	****; P = <0.0001	****; P = <0.0001	**; P = 0.0053	****; P = <0.0001
826-Qβ_implant_W6	***; P = 0.0004	***; P = 0.0003	****; P = <0.0001	ns; P = 0.0534	****; P = <0.0001	*; P = 0.0138

IgG binding to Omicron (B1.1.529) S protein

Tukey's multiple comparisons test (one-way ANOVA) - Week 16, Statistically significant difference and P value

	826-CPMV_100	826-CPMV_200	826-CPMV_implant	826-Qβ_100	826-Qβ_200	826-Qβ_implant	Pre-bleed
826-CPMV_100	N/A	ns; P = 0.6533	ns; P = 0.1338	***; P = 0.0003	****; P = <0.0001	ns; P = 0.0777	****; P = <0.0001
826-CPMV_200		N/A	ns; P = 0.9966	*; P = 0.0453	****; P = <0.0001	ns; P = 0.9778	****; P = <0.0001
826-CPMV_implant			N/A	ns; P = 0.3418	****; P = <0.0001	ns; P = >0.9999	****; P = <0.0001
826-Qβ_100				N/A	****; P = <0.0001	ns; P = 0.4959	****; P = <0.0001
826-Qβ_200					N/A	****; P = <0.0001	****; P = <0.0001
826-Qβ_implant						N/A	****; P = <0.0001
Pre-bleed							N/A

Tukey's multiple comparisons test (one-way ANOVA) - Week 50, Statistically significant difference and P value

	826-CPMV_100	826-CPMV_200	826-CPMV_implant	826-Qβ_100	826-Qβ_200	826-Qβ_implant	Pre-bleed
826-CPMV_100	N/A	ns; P = 0.8737	ns; P = 0.6072	ns; P = 0.9988	****; P = <0.0001	ns; P = 0.8883	****; P = <0.0001
826-CPMV_200		N/A	*; P = 0.0276	ns; P = 0.3355	****; P = <0.0001	ns; P = >0.9999	****; P = <0.0001
826-CPMV_implant			N/A	ns; P = 0.9846	****; P = <0.0001	*; P = 0.03	**; P = 0.0053
826-Qβ_100				N/A	****; P = <0.0001	ns; P = 0.3547	***; P = 0.0002
826-Qβ_200					N/A	****; P = <0.0001	****; P = <0.0001
826-Qβ_implant						N/A	****; P = <0.0001
Pre-bleed							N/A

Tukey's multiple comparisons test (one-way ANOVA) - Comparison between Weeks 16 and 50, Statistically significant difference and P value

	826-CPMV_100_W50	826-CPMV_200_W50	826-CPMV_implant_W50	826-Qβ_100_W50	826-Qβ_200_W50	826-Qβ_implant_W50
826-CPMV_100_W6	****; P = <0.0001	***; P = 0.0002	****; P = <0.0001	****; P = <0.0001	ns; P = 0.7276	***; P = 0.0002
826-CPMV_200_W6	***; P = 0.0006	*; P = 0.0395	****; P = <0.0001	****; P = <0.0001	*; P = 0.0168	*; P = 0.0364
826-CPMV_implant_W6	**; P = 0.0083	ns; P = 0.311	****; P = <0.0001	***; P = 0.0008	**; P = 0.0013	ns; P = 0.2934
826-Qβ_100_W6	ns; P = 0.8471	ns; P = >0.9999	*; P = 0.024	ns; P = 0.3051	****; P = <0.0001	**; P = 0.0016
826-Qβ_200_W6	****; P = <0.0001	****; P = <0.0001	****; P = <0.0001	****; P = <0.0001	***; P = 0.0003	***; P = 0.0006
826-Qβ_implant_W6	*; P = 0.0159	ns; P = 0.4587	****; P = <0.0001	ns; P = >0.9999	****; P = <0.0001	ns; P = 0.4369

Table S5. Two-way ANOVA for Figure 5B using pairwise multiple comparison followed by Tukey's multiple comparison test was used to compare between groups. Asterisks indicate significant differences between groups (*p < 0.05; **p < 0.01, **** p <0.0001).

Avidity index of SARS-CoV-2 S protein IgG

Tukey's multiple comparisons test - Week 6, Statistically significant difference and P value

	826-CPMV_100	826-CPMV_200	826-CPMV_implant	826-Qβ_100	826-Qβ_200	826-Qβ_implant	Pre-bleed
826-CPMV_100	N/A	ns; P = 0.3136	ns; P = 0.336	***; P = 0.0002	***; P = 0.0006	****; P = <0.0001	****; P = <0.0001
826-CPMV_200		N/A	ns; P = >0.9999	**; P = 0.0072	*; P = 0.0243	***; P = 0.0006	****; P = <0.0001
826-CPMV_implant			N/A	**; P = 0.0067	*; P = 0.0223	***; P = 0.0005	****; P = <0.0001
826-Qβ_100				N/A	ns; P = 0.9874	ns; P = 0.6478	**; P = 0.0051
826-Qβ_200					N/A	ns; P = 0.2772	**; P = 0.0016
826-Qβ_implant						N/A	ns; P = 0.0793
Pre-bleed							N/A

Tukey's multiple comparisons test - Week 16, Statistically significant difference and P value

	826-CPMV_100	826-CPMV_200	826-CPMV_implant	826-Qβ_100	826-Qβ_200	826-Qβ_implant	Pre-bleed
826-CPMV_100	N/A	ns; P = 0.3136	ns; P = 0.336	***; P = 0.0002	***; P = 0.0006	****; P = <0.0001	****; P = <0.0001
826-CPMV_200		N/A	ns; P = >0.9999	**; P = 0.0072	*; P = 0.0243	***; P = 0.0006	****; P = <0.0001
826-CPMV_implant			N/A	**; P = 0.0067	*; P = 0.0223	***; P = 0.0005	****; P = <0.0001
826-Qβ_100				N/A	ns; P = 0.9874	ns; P = 0.6478	**; P = 0.0051
826-Qβ_200					N/A	ns; P = 0.2772	**; P = 0.0016
826-Qβ_implant						N/A	ns; P = 0.0793
Pre-bleed							N/A

Tukey's multiple comparisons test - Week 50, Statistically significant difference and P value

	826-CPMV_100	826-CPMV_200	826-CPMV_implant	826-Qβ_100	826-Qβ_200	826-Qβ_implant	Pre-bleed
826-CPMV_100	N/A	ns; P = 0.3136	ns; P = 0.336	***; P = 0.0002	***; P = 0.0006	****; P = <0.0001	****; P = <0.0001
826-CPMV_200		N/A	ns; P = >0.9999	**; P = 0.0072	*; P = 0.0243	***; P = 0.0006	****; P = <0.0001
826-CPMV_implant			N/A	**; P = 0.0067	*; P = 0.0223	***; P = 0.0005	****; P = <0.0001
826-Qβ_100				N/A	ns; P = 0.9874	ns; P = 0.6478	**; P = 0.0051
826-Qβ_200					N/A	ns; P = 0.2772	**; P = 0.0016
826-Qβ_implant						N/A	ns; P = 0.0793
Pre-bleed							N/A

Avidity index of Omicron (B1.1.529) S protein IgG

Tukey's multiple comparisons test - Week 6, Statistically significant difference and P value

	826-CPMV_100	826-CPMV_200	826-CPMV_implant	826-Qβ_100	826-Qβ_200	826-Qβ_implant	Pre-bleed
826-CPMV_100	N/A	ns; P = 0.4255	ns; P = 0.4724	* ; P = 0.0197	**; P = 0.0036	*; P = 0.0125	**; P = 0.0033
826-CPMV_200		N/A	ns; P = >0.9999	ns; P = 0.4647	ns; P = 0.107	ns; P = 0.3313	ns; P = 0.098
826-CPMV_implant			N/A	ns; P = 0.4183	ns; P = 0.0927	ns; P = 0.2938	ns; P = 0.0848
826-Qβ_100				N/A	ns; P = 0.9341	ns; P = >0.9999	ns; P = 0.918
826-Qβ_200					N/A	ns; P = 0.9843	ns; P = >0.9999
826-Qβ_implant						N/A	ns; P = 0.9779
Pre-bleed							N/A

Tukey's multiple comparisons test - Week 16, Statistically significant difference and P value

	826-CPMV_100	826-CPMV_200	826-CPMV_implant	826-Qβ_100	826-Qβ_200	826-Qβ_implant	Pre-bleed
826-CPMV_100	N/A	ns; P = 0.4255	ns; P = 0.4724	* ; P = 0.0197	**; P = 0.0036	*; P = 0.0125	**; P = 0.0033
826-CPMV_200		N/A	ns; P = >0.9999	ns; P = 0.4647	ns; P = 0.107	ns; P = 0.3313	ns; P = 0.098
826-CPMV_implant			N/A	ns; P = 0.4183	ns; P = 0.0927	ns; P = 0.2938	ns; P = 0.0848
826-Qβ_100				N/A	ns; P = 0.9341	ns; P = >0.9999	ns; P = 0.918
826-Qβ_200					N/A	ns; P = 0.9843	ns; P = >0.9999
826-Qβ_implant						N/A	ns; P = 0.9779
Pre-bleed							N/A

Tukey's multiple comparisons test - Week 50, Statistically significant difference and P value

	826-CPMV_100	826-CPMV_200	826-CPMV_implant	826-Qβ_100	826-Qβ_200	826-Qβ_implant	Pre-bleed
826-CPMV_100	N/A	ns; P = 0.4255	ns; P = 0.4724	* ; P = 0.0197	**; P = 0.0036	*; P = 0.0125	**; P = 0.0033
826-CPMV_200		N/A	ns; P = >0.9999	ns; P = 0.4647	ns; P = 0.107	ns; P = 0.3313	ns; P = 0.098
826-CPMV_implant			N/A	ns; P = 0.4183	ns; P = 0.0927	ns; P = 0.2938	ns; P = 0.0848
826-Qβ_100				N/A	ns; P = 0.9341	ns; P = >0.9999	ns; P = 0.918
826-Qβ_200					N/A	ns; P = 0.9843	ns; P = >0.9999
826-Qβ_implant						N/A	ns; P = 0.9779
Pre-bleed							N/A
